# Noise and biases in genomic data may underlie radically different hypotheses for the position of Iguania within Squamata

**DOI:** 10.1371/journal.pone.0202729

**Published:** 2018-08-22

**Authors:** Nicolás Mongiardino Koch, Jacques A. Gauthier

**Affiliations:** 1 Department of Geology and Geophysics, Yale University, New Haven, Connecticut, United States of America; 2 Yale Peabody Museum of Natural History, New Haven, Connecticut, United States of America; Universitetet i Bergen, NORWAY

## Abstract

Squamate reptiles are a major component of vertebrate biodiversity whose crown-clade traces its origin to a narrow window of time in the Mesozoic during which the main subclades diverged in rapid succession. Deciphering phylogenetic relationships among these lineages has proven challenging given the conflicting signals provided by genomic and phenomic data. Most notably, the placement of Iguania has routinely differed between data sources, with morphological evidence supporting a sister relationship to the remaining squamates (Scleroglossa hypothesis) and molecular data favoring a highly nested position alongside snakes and anguimorphs (Toxicofera hypothesis). We provide novel insights by generating an expanded morphological dataset and exploring the presence of phylogenetic signal, noise, and biases in molecular data. Our analyses confirm the presence of strong conflicting signals for the position of Iguania between morphological and molecular datasets. However, we also find that molecular data behave highly erratically when inferring the deepest branches of the squamate tree, a consequence of limited phylogenetic signal to resolve this ancient radiation with confidence. This, in turn, seems to result from a rate of evolution that is too high for historical signals to survive to the present. Finally, we detect significant systematic biases, with iguanians and snakes sharing faster rates of molecular evolution and a similarly biased nucleotide composition. A combination of scant phylogenetic signal, high levels of noise, and the presence of systematic biases could result in the misplacement of Iguania. We regard this explanation to be at least as plausible as the complex scenario of convergence and reversals required for morphological data to be misleading. We further evaluate and discuss the utility of morphological data to resolve ancient radiations, as well as its impact in combined-evidence phylogenomic analyses, with results relevant for the assessment of evidence and conflict across the Tree of Life.

## Introduction

Squamate reptiles (hereafter referred to as lizards, including snakes and amphisbaenians) constitute a major component of vertebrate biodiversity. With 10,336 described species as of March 2018 [1], Squamata constitutes one of the main radiations of extant Tetrapoda, and has been a dominant element in terrestrial ecosystems since at least the Early Cretaceous ([2–4] and references therein). With a long history of comparative research stretching back to the early 19^th^ century, lizards have been elevated to the status of model organisms in fields as disparate as behavior, ecology, functional morphology, biogeography and developmental biology ([5–8] and references therein). Likewise, their unique evolutionary history has inspired theoretical and practical innovations in many topics within evolutionary biology, such as species delimitation [9], ancestral state reconstruction [10, 11], extinction patterns [12], and adaptive radiations [13].

In sharp contrast with the ecological and taxonomic diversity of Squamata today, its lepidosaurian sister clade is represented by a single extant species: *Sphenodon punctatus*, the New Zealand tuatara [14]. This was not always the case, however, as the situation was reversed for much of the Mesozoic, when rhynchocephalians predominated (e.g., [15–17] and references therein). Early representatives of the *S*. *punctatus* total clade were diverse and disparate, globally distributed and ecologically dominant throughout the Triassic and Jurassic [18–20]. Sphenodontian rhynchocephalians are, moreover, abundant in Late Triassic localities from which lizards have yet to be recovered (e.g., [21]). *Sphenodon punctatus* is but a relict of an ancient divergence from the lizard total clade that took place by at least ~240 Ma (Anisian; Middle Triassic; [22]), when most of the early rhynchocephalian radiation had apparently already occurred [23]. Only one potential stem lizard, *Megachirella wachtleri* (~240 Ma; Anisian; Middle Triassic), has so far been identified from the Triassic and Early Jurassic [24]. The crown lepidosaur divergence could have occurred even earlier [24], as representatives of the lepidosaur total clade (Pan-Lepidosauria), such as *Sophineta cracoviensis* [25], are known from ~245 Ma (late Olenekian, Early Triassic), and the oldest fossil referred to Pan-Archosauria, *Aenigmastropheus parringtoni* [26], is about 265 million years old (Capitanian, Middle Permian).

A literal reading of the fossil record indicates that lizards rose to dominance as part of the Early Cretaceous Terrestrial Revolution along with the decline (at least in Laurasia [27, 28]) of sphenodontian rhynchocephalians. Stem-members of several of the major crown squamate clades are known from the Late Jurassic [29], but they are rare in comparison to coeval sphenodontians [15]. In stark contrast, recent molecular estimates suggest that squamate origin and diversification occur very close to the end-Triassic extinction [23, 30–32], coinciding with major changes in climate and vegetation [33, 34]. Even though reconciling molecular and paleontological evidence in order to precisely time the divergence of the main lineages of crown lizards has proven challenging [23], one pattern seems common to most studies performing temporal inferences: the main lineages within crown Squamata diverged in a very short time span a long time ago. All major surviving clades of lizards (Anguimorpha, Dibamidae, Gekkota, Iguania, Lacertoidea, Scincoidea and Serpentes) seem to have originated in rapid succession, spanning an interval of time estimated to range between 23.1–43.5 Ma [23, 30–32, 35]. This means that the short internodes connecting the major clades that constitute the backbone of the squamate tree have lengths that are on an average of only 4.6–8.7 Ma (S1 Table).

Ever since the first explicit phylogenetic analyses of Squamata ([36], see also [37]), most studies based on morphological evidence have found unambiguous support for a basal split between Iguania and all remaining lizards [29, 38–41], a taxon for which Estes et al. [36] coined the name Scleroglossa. The name of this clade derives from the flattened and keratinized tongue that characterizes all its members, which contrasts with the fleshy, highly muscular tongue of both iguanians and *Sphenodon punctatus* [36, 42, 43]. This transition in tongue morphology signals a major shift in foraging mode from the visually oriented, sit-and-wait strategy of non-scleroglossan lepidosaurs, that capture their prey using lingual prehension (a technique epitomized by chameleons), to the active hunting style of scleroglossans that rely more on chemoreception to target prey, and use only their jaws to secure them [44]. Under this evolutionary scenario, the release of the tongue from its predatory role facilitated further elaboration of its vomero-olfactory function. As a consequence, a large set of nested evolutionary innovations relating to the morphology of the tongue, the vomeronasal organ and the adjacent cranial elements, is evident as one traverses the scleroglossan tree leading to crown caenophidian snakes [29, 42, 45–48].

The foundations of this taxonomy, as well as its evolutionary implications, were undermined with the advent of molecular phylogenetics. Although the first molecular approaches based on mitochondrial genes were unable to resolve confidently the oldest branches within Squamata [49, 50], later attempts using nuclear genes found support for a highly nested position of Iguania, closely allied with snakes and anguimorphs [51–56]. The clade uniting these three lineages was named Toxicofera due to the shared expression of toxin genes in their salivary glands [57], suggesting an early origin of venom [58]. This conclusion was overturned by subsequent research indicating that these genes are likely to have housekeeping functions, and are expressed in multiple tissues by species both inside and outside Toxicofera [59, 60]. Furthermore, this topology requires that the similarities between iguanians and *Sphenodon punctatus* are the product of a complex history of reversals and convergences, implying astonishing levels of homoplasy among morphological characters [29, 36, 38, 46]. Nonetheless, this alternative position for Iguania received further support as the field transitioned into phylogenomics, with Toxicofera being found across datasets based on both transcriptomes [61] and ultra-conserved elements (UCEs) [62].

In light of these results, many have favored the molecular topology, assuming that the misleading signal must come from morphology [63–66]. As explained by Wiens & Lambert [67], the decision to accept Toxicofera as the best depiction of lizard interrelationships is warranted based on two lines of evidence: the same higher-level structure is generally supported in studies combining molecular and morphological evidence, and phenotypic data have been shown to harbor misleading signal. The latter refers to the fact that morphological evidence often unites all or most long-bodied, limb-reduced, head-first burrowing lizards into a single (poorly supported) clade, when there is ample evidence that fossorial habits, body elongation and limb reduction/loss has in fact occurred multiple times independently [64, 65]. Using this same logic, a recent publication claimed to have solved the conflict over deep-time relationships among lizards [56], a claim that received further support from the first study in which Toxicofera was inferred based on morphological apomorphies alone [24].

While a proper response to these claims is beyond the scope of this paper, we note that both studies pose significant problems. For example, all six of the unambiguous apomorphies said to diagnose Toxicofera [56], are not in fact present in Iguania, and therefore must reverse to their ancestral conditions in that clade [29]. Reversals can be consistent with a tree, but the topology of that tree depends fundamentally on there being a sufficient number of un-reversed apomorphies supporting it. Thus, the six toxicoferan apomorphies only seem unambiguous because their uniformly contrary message was overridden by more numerous molecular characters, not because morphology actually supports a nested position for Iguania on its own merits [29]. The recent study arguing that Gekkota, rather than Iguania, is sister to all other squamates [24] could serve as a stronger case in that several (poorly supported) trees inferring that topology were based exclusively on morphology. That extraordinary claim is, however, more difficult to evaluate as Simões et al. [24] did not explicitly identify (much less figure or discuss) any apomorphies relevant to relations among crown squamates.

Many have expressed doubts that this matter is settled [29, 31, 44, 45, 68]. Some authors have even chosen to continue employing a scleroglossan phylogenetic framework [69, 70], while others have used both topologies to explore the evolutionary history of novel characters and particular sub-taxa [71–75], further indicating that a consensus has yet to be reached. Although phenotypic convergence has routinely been proposed to explain this conflict—given the similarity in feeding habits shared by *Sphenodon punctatus* and iguanians—the diversity of anatomical regions, in disparate ecological and functional contexts, and embryological origins for the morphological synapomorphies supporting Scleroglossa implies that this likely constitutes a simplistic explanation [29, 46]. Further research has also shown that toxicoferan monophyly is not only statistically rejected by cranial morphological data (as would be expected in a scenario in which the convergent evolution of lingual prehension is the source of the misleading signal), but also by data from vertebral and, more generally, postcranial morphology [56]. Furthermore, although combining evidence has been justified on philosophical grounds [76], as well as considered a method that maximizes explanatory power [77, 78], it is equally true that combining conflicting datasets can produce misleading results [79–82]. Finally, there have been suggestions that the erroneous signal might come from the molecular dataset, with possible confounding factors including the extreme distance to the outgroup [68], the heterogeneity in rates of molecular evolution across clades [83], and the extremely short and deep internodes that constitute the backbone of the lizard tree [45]. We provide novel insights into this problem by generating an expanded morphological dataset and performing new analyses in order to explore the presence of phylogenetic signal, noise and biases in the molecular data.

## Results

We assembled an expanded morphological dataset consisting of 848 characters (S1 File), of which 165 were treated as ordered and the remainder were left unordered. This represents an increase of roughly 11% with respect to the largest morphological dataset for the clade published to date. The majority of characters were taken from Gauthier et al. [29], with modifications proposed by Hsiang et al. [71] and Longrich et al. [41], and with significant contributions of external morphological characters from Reeder et al. [56]. The molecular matrix on the other hand is the same as used by Reeder et al. [56], and is composed of sequence data from 46 protein-coding genes (S2 File).

In order to assess the placement of the main clades comprising the squamate ‘backbone’ (namely: taxa traditionally assigned to Anguimorpha, Gekkota, Iguania, Lacertoidea, Scincoidea, and Serpentes) in both molecular and morphological datasets, we had to restrict taxon sampling. First, we only considered extant taxa represented in both datasets, leading to the exclusion of all fossils, including potentially influential backbone clades such as Polyglyphanodontia and Mosasauria, the critically important stem squamatans (*Huehuecuetzpalli mixtecus* and *Megachirella wachtleri*), as well as less modified members of the rhynchocephalian outgroup. Second, we excluded all serpentiform taxa with the exception of Serpentes (hereafter = snakes) as the repeated evolution of a snake-like habitus has confounded morphological analyses since at least Cuvier’s day (e.g., [84]). This prevents us from considering the phylogenetic position of a number of enigmatic clades, most notably Dibamidae, that have proven problematic for both types of data. Other snake-like taxa are well nested within the main clades included in all datasets, so their exclusion is not expected to bias our phylogenetic results. In the end, we retained 46 species for all analyses as they largely conserve the same topological relationships to one another seen in more comprehensive analyses while maximizing coverage within the aforementioned clades. Although the benefits of increased taxon sampling have been proven repeatedly [85–88], the backbone topology for lizards, while differing markedly between data sources, is nonetheless remarkably stable within them across a wide range of taxon sampling [68]. So, we do not expect our restricted sample to affect significantly our results, especially as this potential shortcoming is largely irrelevant to the question being addressed here: the radically different hypotheses for the position of Iguania within Squamata.

**Fig 1:**
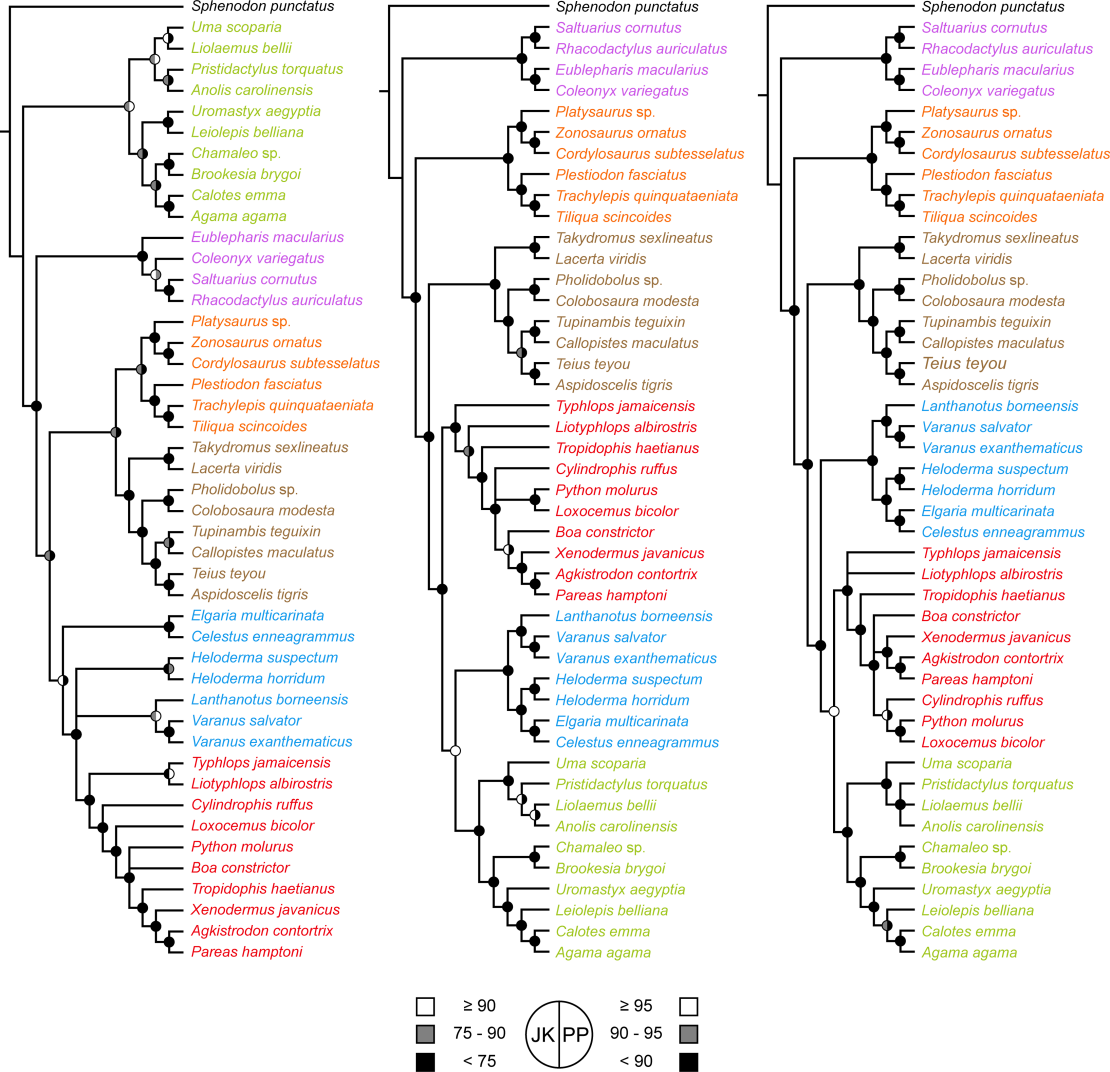
**Summary of phylogenetic relationships obtained using morphological (left), molecular (center) and combined datasets (right)**. Topologies correspond to the strict consensus of the optimal trees under equal-weights maximum parsimony and Bayesian inference for each dataset (original trees can be found in S4 File). Main lizard clades are color coded: light blue = Anguimorpha, purple = Gekkota, green = Iguania, brown = Lacertoidea, orange = Scincoidea, red = Serpentes. Circles on nodes correspond to support values, coded as shown at the lower left corner. JK = jackknife, PP = posterior probability.

The molecular and morphological phylogenies (Fig 1 and S1–S6 Figs) proved fully congruent with the results of previous studies based on those data sources (but see [24]). Molecular data strongly support the successive branching of Gekkota, Scincoidea, Lacertoidea, and Toxicofera (which includes Iguania), but with weak support for the resolution of the main clades within the latter. In contrast, the morphological phylogeny differs by strongly supporting a first split between Iguania and Scleroglossa, as well as by uniting Scincoidea with Lacertoidea in a clade (= Scincomorpha Camp 1923 as that name was defined by Estes et al. [36]). Synapomorphies supporting this deeper structure are not concentrated in any single anatomical module, but are rather drawn from a range of morpho-functional systems (S3 File). As in previous analyses, Serpentes is nested inside Anguimorpha, being more closely related to Varanoidea than to Anguidae. Beyond the starkly conflicting placements among backbone taxa, relatively few differences are found in topologies inferred inside these major clades, and these are generally restricted to nodes with low support values in either one or both the morphological and molecular analyses (e.g., the relationships among main subclades of Iguanidae and Acrodonta, the internal resolution of Gekkota, as well as a few problematic nodes within snakes). In fact, as highlighted previously [31], the alternative topologies are highly congruent, with an SPR similarity in the range of 0.77–0.79. This similarity further increases to 0.81–0.84 if poorly supported nodes are collapsed, leaving most incongruence restricted to the resolution of the earliest nodes in the tree. However modest, this incongruence is statistically significant, as determined by Templeton [89], ILD [90], and AU [91] tests (*P* < 0.01 in all cases).

As previously shown for lizards [56, 92], the combined tree is not identical to the topology derived from molecular data alone. This confirms that adding a comparatively small morphological dataset can still have an impact in combined analyses (see also [71]). However, all differences are restricted to nodes with limited support in the molecular analyses. Some studies have reported that the signal from morphology can overturn nodes strongly supported by phylogenomic datasets [92]. In our study, however, we found that all nodes that are resolved differently after the addition of morphological data are present in less than 50% of the gene trees (S7 Fig), and that the resolution obtained in the combined-evidence analysis is always among the alternatives present in the set of gene trees (S2 Table). This suggests that the role of morphology in studies employing phylogenomic datasets might be restricted to shifting the balance between alternative topologies already supported by molecular data, independent of whether the conflicting molecular signal is evident or masked by high support values [93, 94].

**Fig 2:**
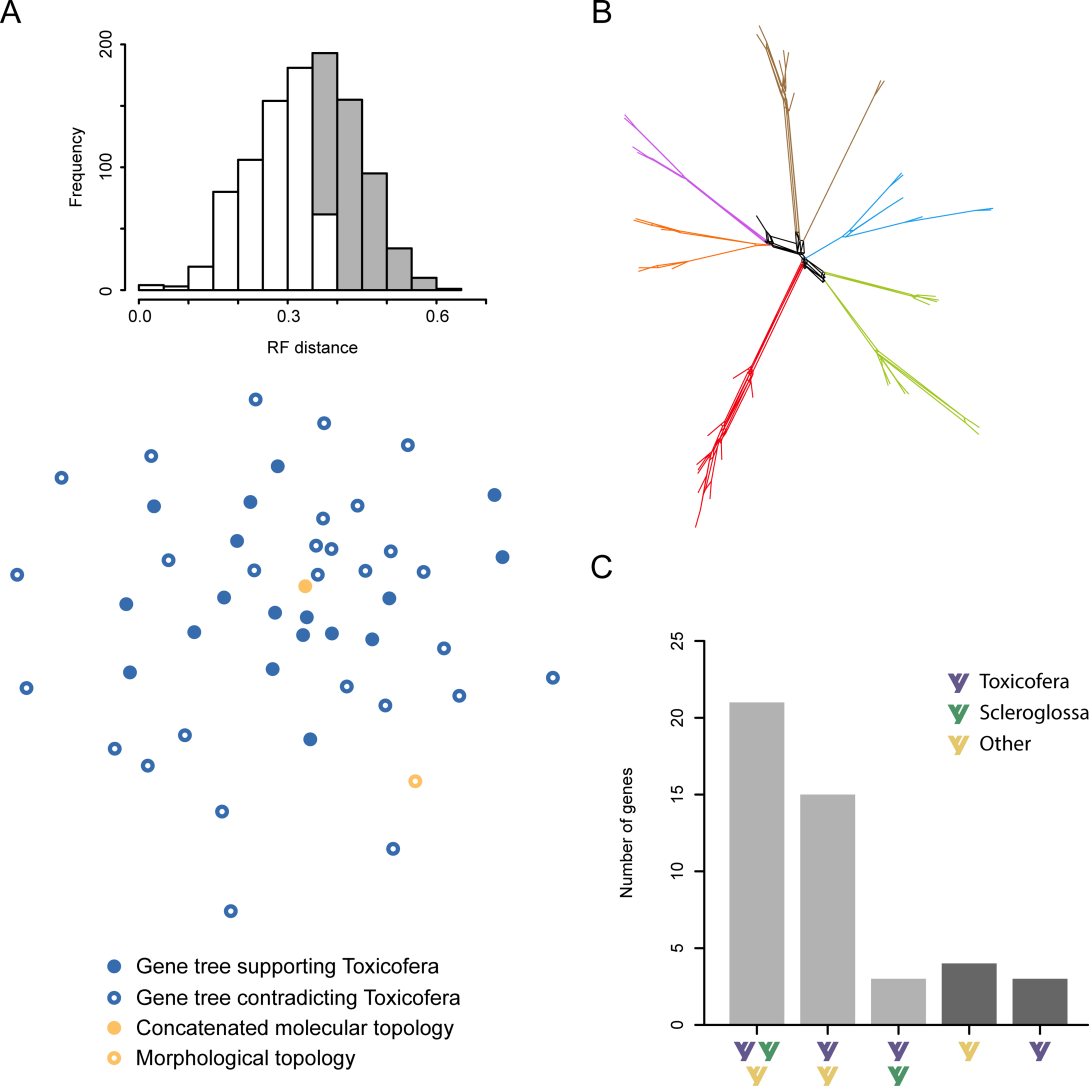
Incongruence in the inference of the lizard tree based on individual genes. (A) Projection of topological differences among gene trees on a two-dimensional treespace. The histogram shows the distribution of pairwise Robinson-Foulds (RF) distances among gene trees, with grey bars representing the fraction of distances larger than the that between the morphological and concatenated molecular topologies (yellow circles). (B) Supernetwork condensing gene tree incongruence. Clades are colored as in Fig 1. Most topological variability is restricted to the backbone (black branches), characterized by different resolutions (reticulations) found at a low frequencies (C) Histogram showing the types of topologies found in the confidence set of trees for all genes. Light grey bars show numbers of genes for which the confidence set contains more than one option (85%), revealing insufficient levels of phylogenetic signal to distinguish among competing resolutions of the lizard backbone clades.

The analysis of the topological differences across gene trees reveals a surprising degree of conflict in the relationships supported by individual genes (Fig 2A), with the morphological topology lying within the region of treespace occupied by molecular data. In fact, the Robinson-Foulds distance (RF distance; the sum of bipartitions in each tree not present in the other, as a fraction of the total number of bipartitions present in both) between the concatenated molecular and morphological trees is very similar to the average distance between pairs of gene trees (see histogram of Fig 2A). This reveals that the topological incongruence found when comparing different molecular estimates of phylogeny is of similar magnitude to that found when comparing molecular data against morphology. Further exploration of gene trees (available as S4 File) indicates that most of this incongruence is restricted to the earliest branches of the lizard tree, as well as to some undisputed higher-level clades such as Lacertoidea, Scincoidea and Iguania, which are not inferred to be monophyletic in 46%, 41% and 26% of gene trees, respectively. This uncertainty in the pattern of early branching is especially clear when split frequencies are used to build a supernetwork (Fig 2B), revealing that gene-tree disagreement almost entirely stems from difficulties with inferring interrelationships among the major clades of lizards, as well as some early divergences within them (S7 Fig).

Multiple factors could explain this pattern of incongruence. Different genes can support different topologies when they have experienced different evolutionary histories, a phenomenon that can occur through the process of incomplete lineage sorting (ILS). This has been considered a possibility given the short internode lengths at the base of the lizard tree [54, 56]. The impact of ILS has, however, been demonstrated to contribute a minimal fraction of the total phylogenetic conflict for deep divergences [95–97]. Simulations have also found maximum parsimony (MP) to succeed at recovering the correct species tree even in the presence of ILS [98], while the backbone divergences in our MP and Bayesian inference (BI) trees inferred from the concatenated molecular dataset are identical. Alternatively, phylogenetic inference can also be led astray when relatively few characters are sampled (i.e., due to sampling error), or when phylogenetic signal is too weak to confidently resolve a particular set of nodes. In the first of these two scenarios, recovering the true tree is expected as character sampling increases, although this might not be the case if characters lack sufficient information. In support of the first of these two scenarios, Reeder et al. [56] argued that genes supporting Toxicofera are significantly longer, and therefore are expected to be less affected by sampling biases, than those rejecting this clade. Nonetheless, gene-alignment length is a product of both the true physical length in the genome (i.e., the number of sampled nucleotide positions, presumed to be independent replicates of the evolutionary process) and the procedure used to obtain a multiple-sequence alignment (MSA). The use of three different methods to assess MSA quality demonstrates that the longer genes in this dataset are in fact significantly enriched in poorly-aligned positions (linear regression between the total number of columns in the MSA of a gene and the proportion of columns eliminated by trimAl [99], BMGE [100], and Gblocks [101]; *P* = 0.044, 0.029 and 0.045, respectively; S8 Fig). Therefore, it seems that the set of genes supporting Toxicofera is unlikely to be enriched in loci that have overcome sampling errors, but are instead enriched in genes with uncertain alignments and poorly supported primary homologies.

In order to specifically test whether the high levels of gene tree discordance affecting the resolution of the lizard backbone topology is a consequence of limited phylogenetic signal, we statistically compared alternative topologies using AU tests [91]. Using a series of monophyly constraints, we tested the ability of each gene to distinguish between alternative hypotheses that support scleroglossan vs. toxicoferan monophyly, or simultaneously contradict both. Our results show that at least two of these options are included in the confidence set of trees for 85% of the genes (Fig 2C), confirming that most of the genes in the molecular dataset offer levels of phylogenetic signal too low to distinguish statistically between alternative resolutions of the most ancient divergences among lizards. Furthermore, of the remaining seven genes, four statistically reject a Toxicofera-bearing topology, with significant phylogenetic signal for this clade restricted to just three genes (or about 6.5% of all molecular data).

**Fig 3:**
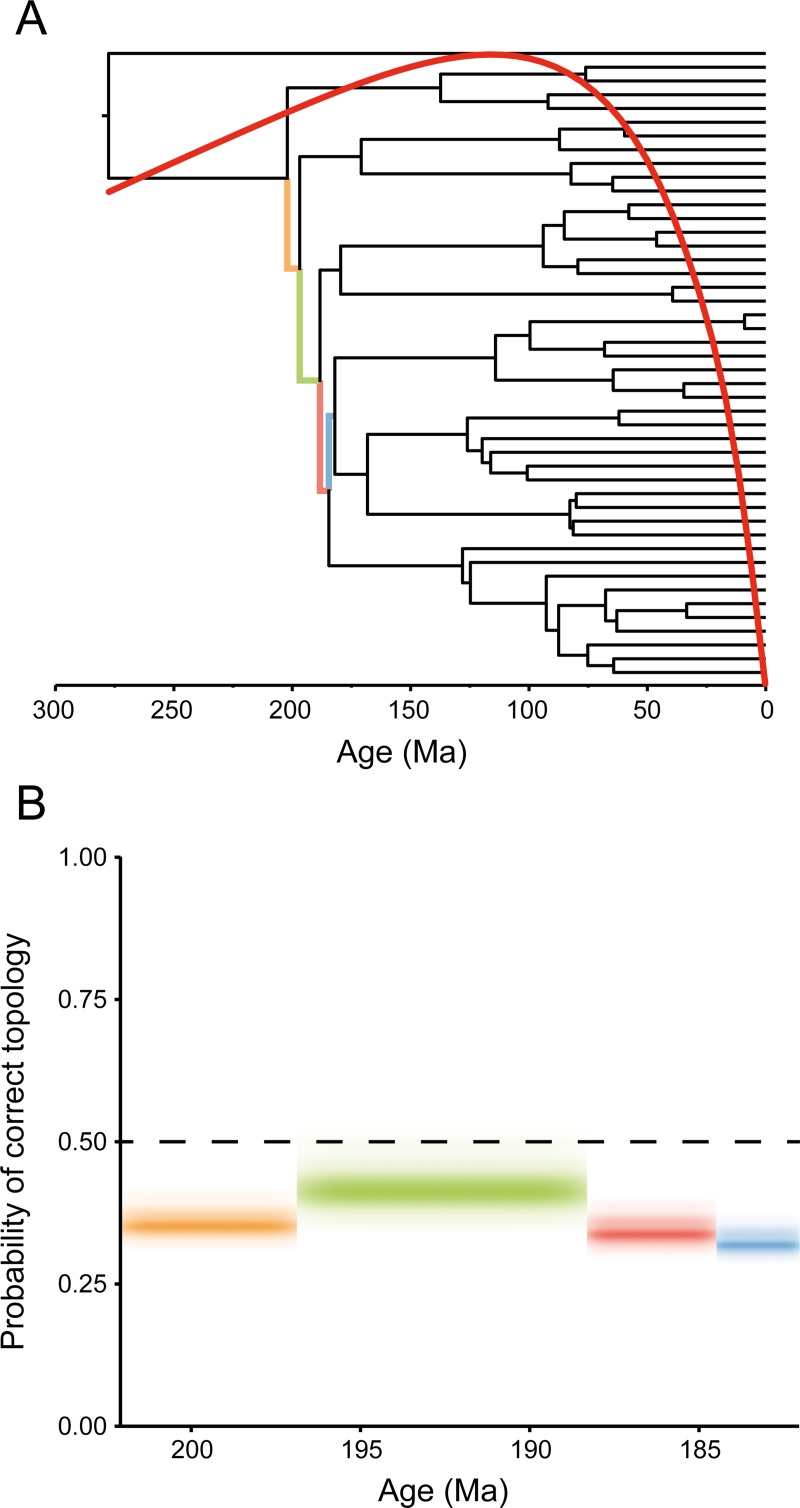
Analysis of the rate of evolution in the molecular dataset. (A) Time-calibrated topology of Zhen & Wiens (2016) with the PI profile of the molecular dataset superimposed (red curve). The informativeness of the dataset decays by the time spanned by the initial crown squamate radiation (branches highlighted in color). (B) Signal and noise analysis of individual genes. The y-axis represents the probability with which individual genes contribute to the correct resolution of the quartets centered on each of the four backbone branches (colored as in A). The stronger the color, the more likely a given probability-outcome is in the set of genes analyzed.

Phylogenetic informativeness (PI) profiles seem to further support the idea that gene tree incongruence derives from low levels of phylogenetic signal, revealing an informativeness peak for the molecular dataset at around 116 Ma and a decay of between 6.5 and 9.2% at the time spanned by the initial crown-squamate radiation (Fig 3A). The analysis of individual PI profiles per gene show similar patterns, with the vast majority of genes displaying profiles that peak at much younger times than those corresponding to the timespan of interest (S9 Fig). Genes that support toxicoferan monophyly do not peak at ages significantly different from those of genes rejecting it (one-way ANOVA: F_1,44_ = 2.42, *P* = 0.13) and, therefore, cannot be considered a subset better disposed to resolving deeper nodes. However, by not considering the detrimental effects of homoplasy, this method is expected to offer only a conservative estimate when branches are short and evolutionary rates are high [102]. To better estimate the potential impact of this decay, a signal and noise analysis was performed to estimate the probabilities of correct, incorrect and polytomous resolutions of the four internodes determining relationships among the major lizard clades [103]. The results indicate that rates of molecular evolution are too high to enable accurate resolution of these four short and ancient branches (Fig 3B). Across all genes employed, we found high probabilities of incorrect resolution for each of those four internodes (on average, between 11 and 74% more likely than correct resolutions, S3 Table), stemming from the accumulation of high levels of phylogenetic noise. Given that a monophyletic Toxicofera requires that the first three of these internodes are simultaneously correct, for any given gene tree supporting Toxicofera, the probability that this clade is the product of true phylogenetic signal is less than 0.1.

In order to attempt to mitigate the potential detrimental effects of fast-evolving sites, we performed a set of new maximum likelihood (ML) phylogenetic analyses after excluding different subsets of fast-evolving sites, a practice that has become common when dealing with ancient divergences [104–108]. We used two different tree-independent methods to estimate the rate of evolution of molecular characters [109, 110] and inferred phylogenies after removing progressively larger subsets of fast-evolving sites. Both methods yield the same result: the first topological change observed as fast characters are eliminated from the dataset is the simultaneous collapse of both Toxicofera and Iguania + Anguimorpha nodes (S10 Fig), even when the molecular dataset still contained between 8.5 and 10 thousand parsimony-informative characters, depending on the method employed (see Methods). Further character removal collapses all remaining backbone nodes in the phylogeny inferred. This paradoxical result exemplifies the extent to which the backbone topology of Squamata derived from the molecular dataset is determined by noisy characters that are unlikely to still retain true historical signals.

**Fig 4:**
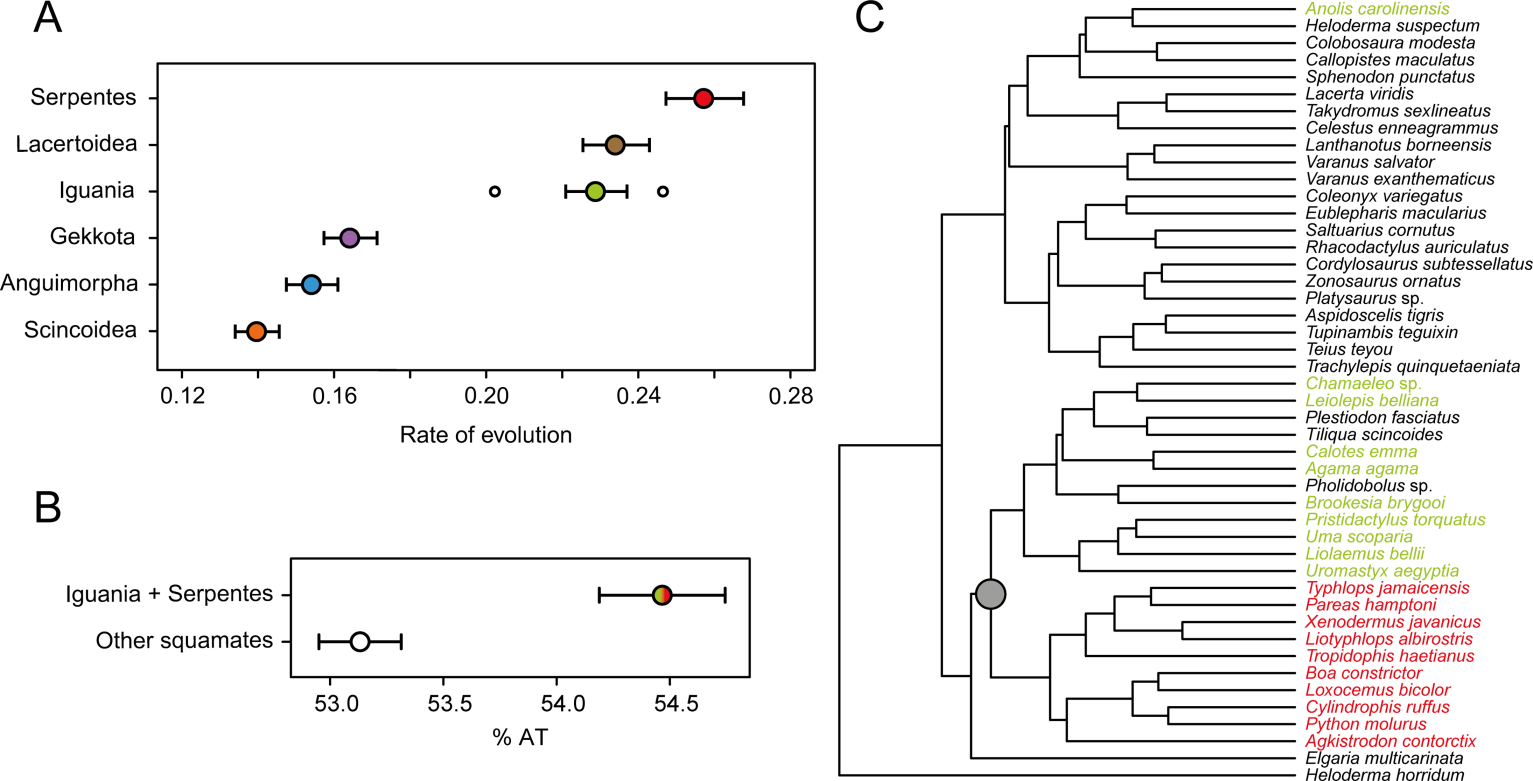
Systematic biases in the molecular dataset. A. Inferred rate of evolution for each of the main clades of lizards studied. Both the median and 95% confidence intervals are represented. In the case of Iguania, the white dots also show the median values for the rate of evolution estimated individually for Acrodonta (right) and Iguanidae (left). B. Nucleotide composition of snakes and iguanians differs systematically from that of the remaining squamates. Values correspond to the average percentage of AT ± 1 standard deviation. C. Clustering of snakes and iguanians (grey dot) due to similar patterns of AT skewness. The tree represents a hierarchical clustering dendrogram, estimated using Euclidean distances of AT skewness per gene.

Additional evidence suggests that the apparent support for a nested position of Iguania might be the result of systematic biases. As already suggested by previous studies [83], we find that both snakes and iguanians (as well as lacertoids), have elevated rates of molecular evolution, about 60% faster than those of the remaining clades (Fig 4A). Furthermore, a chi-square test reveals significant levels of base-frequency heterogeneity (*P* < 10^16^). Detailed exploration of nucleotide use indicates that iguanians and snakes share a genome that is, on average, 1.3% AT-richer than that of the remaining lizard clades (Fig 4B), a difference that is significant under a phylogenetic ANOVA (F = 16.92, *P* = 0.035). Moreover, we find that iguanians and snakes cluster together due to similar patterns of AT skewness across genes (Fig 4C).

**Fig 5:**
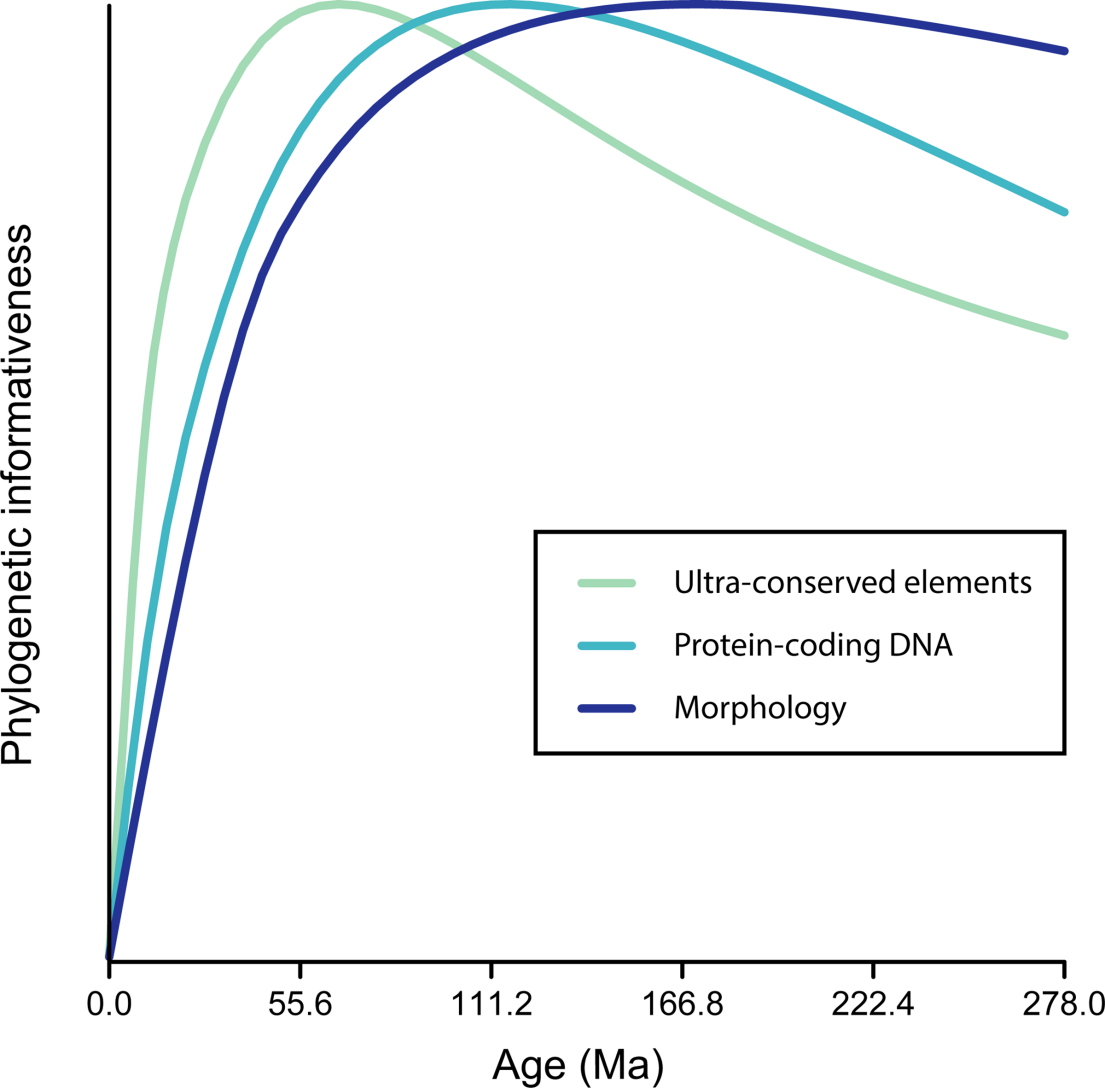
Comparison of the PI profiles for the protein-coding (same as in Fig 3) and UCE datasets with morphology. Morphology evolves at a slower rate, leading to a PI-profile peak at around 171.5 Ma, an age markedly older than either molecular dataset. The rate of decay is also less steep for the morphological data. Thus, morphology accumulates noise at a much slower pace, potentially retaining more phylogenetic signal to resolve ancient and short branches. The height of the profiles is standardized to emphasize their temporal dynamics; when calculated without standardization, peak informativeness is 75% lower for morphology, and 55% lower for UCEs, than is the peak for protein-coding data measured on a per character basis. This standardization is applied in recognition of the fact that morphological characters are preselected to be informative during the timeframe under study; therefore, their absolute informativeness cannot be compared directly, whereas the shape of their informativeness profile—when their rate of change implies that they will be useful for phylogenetic inference—remains of interest.

The presence of branch-length and compositional heterogeneities might result in a misplaced Iguania. For this to be true, however, these biases need to be genome-wide, as Toxicofera has been recovered from a wide variety of coding and non-coding loci (e.g., [56, 61, 62]). To explore this possibility, we further analyzed the phylogenetic signal and compositional spectrum of a molecular dataset consisting of 4,178 squamate UCEs [62]. Once again, we find that iguanians and snakes share a genome that is AT-richer (phylogenetic ANOVA, F = 5.45, *P* = 0.03), even when this dataset includes only three members of each of these clades. Although UCEs have been considered especially suitable for phylogenetic inference in deep-time [111, 112], few studies have characterized their temporal performance (such as [113]). Surprisingly, our results reveal that the phylogenetic informativeness of the squamate UCE dataset peaks at times even younger than that of the protein-coding loci dataset previously analyzed (Fig 5), which in turn shows a shallower maximum informativeness than the morphological dataset. Phylogenetic signal surviving from the initial squamate radiation might be scant irrespective of the type of molecular data employed, while systematic biases seem to be present throughout the genomes of lizards.

## Discussion

The development of methods that allow genome-scale molecular data to be sequenced revolutionized the field of phylogenetic systematics [114]. Like many previous revolutions, it arrived with a renewed faith that the Tree of Life, depicting the true relationships among all organisms, was finally within reach [115, 116]. More than a decade has passed since the first pioneering works in phylogenomics, and although this approach has contributed mightily toward resolving diverse phylogenetic problems [117–122], other regions of the Tree of Life have remained immune to the exponential growth in the amount of data. These include the position of turtles among reptiles [123], the initial radiation of Neoaves [124], the earliest divergences among placental mammals [125], the internal resolution of Chelicerata [126] and Lophotrochozoa [127], and the deepest nodes of animal phylogeny [128]. In all of these cases (as well as several others), the virtual eradication of sampling error through massive sequencing has not proven to be the panacea once hoped for [115].

The elimination of stochastic errors has thus opened a new chapter for phylogenetic inference, in which systematic biases, coupled with the use of models of evolution that are simplistic compared to the complexity of genomic data, are expected to play a central role [129–131]. The fact that alternative phylogenomic analyses built to tackle the same questions arrive at conflicting results as, for example, in the case of birds [132, 133] and metazoans [134, 135], indicate that these problems are sufficiently important to render phylogenomic inference inconsistent. These issues are sometimes difficult to diagnose, as researchers continue to rely on measures of support that are expected to conceal signs of conflict and systematic error [93, 94, 123, 136]. Furthermore, an often unstated assumption is that the characters employed are evolving at a rate appropriate to estimate with accuracy the phylogenetic problem at hand [137]. If this assumption is not met and characters evolve at a rate faster than optimal, true historical signal will be eroded as phylogenetic signal is replaced by random noise [138]. As the phylogenetic signal-to-noise ratio decays, non-phylogenetic signals stemming from processes such as heterotachy and compositional heterogeneity can dominate the analysis, a phenomenon that is unlikely to be overcome by increasing the amount of data through random sampling of the genome [103, 114, 128, 130, 139]. Although there has been significant progress toward detecting and accounting for these phenomena, we are still far from understanding how phylogenetic signal, noise and biases interact to determine the outcome of genome-scale phylogenetic studies [95, 123, 140, 141].

Ancient rapid radiations are expected to be disproportionally affected by these issues [94, 142–144]. Given the bush-like shape of these trees, with short branches close to the base and long ensuing timespans, characters are required to evolve fast enough to capture the radiation as it unfolds, but slow enough that this information is not erased by subsequent evolution [138, 145–147]. Increasing the amount of data by random sampling is not expected to yield many characters that fulfill these prerequisites [142], while rendering analyses more vulnerable to systematic biases [130]. These problems are thought to cause persistent difficulties in resolving many of the aforementioned empirical examples. Further simulation studies have shown that—even under ideal conditions that are never met in the analysis of empirical data—the probability of recovering the true basalmost split for “bushy” trees can be negligible [148].

The phylogeny of Squamata has become a paradigmatic example of character incongruence [46], with morphological and molecular data displaying strong disagreement regarding the position that iguanian lizards occupy [29, 54]. Given this signal incompatibility, many have assumed that morphology must be providing an incorrect estimate of phylogeny [56, 67]. Morphology does indeed have problems resolving some parts of the lizard tree, most conspicuously when inferring relationships of long-bodied, limb-reduced, head-first burrowing lizards [65]. However, this problem should not affect the placement of Iguania, a clade that has never produced any serpentiform taxa. Indeed, our results show that once the potentially confounding factor of independent adoption of a snake-like habitus is excluded, morphological data are capable of providing a well-supported alternative hypothesis for the interrelationships of the main clades of lizards (Fig 1). The proposal that morphology is confounded by apomorphies in the feeding behaviors of Iguania and *Sphenodon punctatus* (interpreted as convergent on the molecular tree) has received little support from a recent study showing that the signal rejecting the position of Iguania within Toxicofera is distributed across cranial and postcranial characters ([56], see also S3 File). Although convergence among these taxa related to feeding behavior remains plausible, it has yet to be firmly established, and at the very least it seems overly simplistic [29, 45, 46, 68].

A second reason why Toxicofera has been favored by molecular systematists is the fact that combined analyses also infer this clade. This justification is based on the idea that, even when morphology represents a minimal fraction of the total dataset, morphological signals still contribute to the result of a combined analysis, altering resolution of even strongly supported nodes [92], a counterintuitive result [149]. Our analyses indicate that the regions of the tree that change with the addition of morphology are all subject to strong conflict within the molecular partition itself, and that the resolution obtained in the combined analysis is always among the options supported by individual gene trees. Although morphology does indeed affect the tree obtained, it seems unable to propose new topologies when it constitutes a minimal fraction of the total evidence. Thus, the role of morphology in the genomic era might be restricted to tipping the balance between alternative resolutions supported by molecular evidence, placing significant limits on its influence in combined analyses.

As expected for an ancient radiation, we find that individual genes harbor very limited phylogenetic signal to resolve interrelationships among the main clades of lizards emerging during the relevant time interval (Fig 2). This result was fully acknowledged in the early days of molecular phylogenetics, when studies still relied on individual genes [49, 50]. This lack of power seems to reflect a rate of molecular evolution that is too high for true historical signals to survive until the present day (Fig 3). The phylogenetic signal–to–noise ratio is low enough that the probability of having greater support for an incorrect topology exceeds that of obtaining the correct resolution across all genes and for all four backbone internodes. Genes supporting Toxicofera do not appear any more reliable than those rejecting it, they do not evolve at significantly different rates, nor do they seem less subject to sampling biases, as previously proposed [56]. In fact, they seem to be genes with low alignment certainty, a property recently found to predict a gene’s lack of reliability for phylogenetic inference [123]. These results suggest that confidence in any given higher-level resolution of the lizard tree, including Toxicofera, is unwarranted. That being said, almost every molecular study relying on more than a handful of genes seems to have converged on similar higher-level topology, a seemingly paradoxical result.

The combination of data derived from multiple genes reduces the impact of sampling errors, therefore increasing the signal-to-noise ratio of a dataset [150]. However, this signal need not result from evolutionary relatedness, as it may also stem from similarities in the processes that shape genomic variation. Intriguingly, we find that snakes and iguanians share both faster rates of molecular evolution and similarly biased nucleotide compositions in their genomes (Fig 4). These potentially confounding factors were initially considered possible causes of the topological incongruence in lizard phylogeny [52], but were discarded owing to a lack of sufficient evidence stemming from small molecular datasets. Other studies have suggested that acrodont iguanians and snakes shared a particularly high rate of molecular evolution [83], which could ultimately influence their inferred relationships [151]. Our results show that although molecular evolution in acrodonts is especially fast, a shift toward higher rates seems to have happened along the branch leading to all iguanians. This could, in turn, affect the position of the entire clade due to long-branch attraction artifacts. Furthermore, both iguanians and snakes seem to share similar values of both GC content and AT skewness. The simultaneous effect of these systematic biases, coupled with a weak phylogenetic signal, might be sufficient to result in a misplaced Iguania. We find evidence for similar issues in a phylogenomic dataset composed of non-coding UCEs [62], showing the aforementioned problems are likely to characterize molecular information genome-wide. Given the complex set of morphological convergences and reversals required for Toxicofera to be true (S3 File), we believe attributing biases to either molecular or morphological data to be equally plausible explanations for the conflict over deep-time squamate relationships, indicating that the conflict over lizard phylogeny is still unresolved.

A comprehensive understanding of evolution requires the integration of genomic and phenomic evidence [152, 153]. Even though morphological data are prone to problems arising from convergent evolution and lack of character independence, it is expected to be relatively free of other types of biases affecting molecular data [150], such as those described above. Morphological evolution is in many respects fundamentally different from evolution at the molecular level [31, 147, 154], and characters are generally preselected to be useful for a given phylogenetic question, all of which might make morphological data especially suited to resolving deep and “bushy” areas of the Tree of Life for which molecular evidence is expected to be scant [31, 149, 155–157]. In fact, the PI profile for our morphological dataset peaks at substantially deeper times than both of the molecular datasets explored (Fig 5), suggesting morphology might provide a more accurate resolution of the initial crown-lizard radiation. A dismissive attitude toward phylogenetic hypotheses based on morphology is therefore not warranted [68, 149, 155, 158, 159]. Many recent publications have employed phylogenomic data to resolve apparent conflicts between morphological and molecular data in favor of topologies originally supported by morphology alone, including the Strepsiptera problem [160], sponge paraphyly [161] and the relationships among the main clades of myriapods [162], copepods [163] and otophysan [95] and siluriform actinopterygians [164]. These examples illustrate that congruence between morphological and molecular evidence is still crucial for phylogenetics [149] and should be sought regardless of the amount of molecular data supporting any given hypothesis.

## Methods

### Datasets and phylogenetic inference

Taxon-character datasets employed can be found as S1 and S2 Files. Throughout, three terminals are identified using generic epithets only, as morphological and molecular data were obtained from different species within those clades. Although novel characters were added exclusively to the morphological dataset, both matrices were modified with respect to previous studies due to the reduction in taxonomic coverage. The molecular matrix contains 619 fewer positions than the equivalent matrix of Reeder et al. [56], all of which were represented entirely by gaps among the 46 terminals selected. Likewise, the number of states for some morphological characters was reduced with respect to those of Gauthier et al. [29], eliminating states not observed among the sampled taxa, thus leading to more accurate estimates of rate matrices [165]. A more complete morphological matrix coded for a wider sample of living and extinct lizards, as well as appropriate character descriptions and illustrations, will be published elsewhere.

Phylogenetic inference was performed on the morphological, concatenated molecular and combined matrices under MP, ML and BI. Because ML phylogenies derived from morphological data failed to support iguanian monophyly, a clade otherwise supported across all methods and analyses, results from ML are not shown. This is in line with recent results suggesting that ML analyses under the Mk model [166] might be the least accurate method of phylogenetic inference from phenotypic data [167].

Parsimony analyses for all datasets were performed in TNT v. 1.5 [168] under equal weights. In all cases, we performed a driven tree search using new technologies [169–171] until the same minimum tree length was found fifty independent times. A round of TBR branch swapping was then performed on the trees in memory. Support was evaluated using absolute node frequency in 1,000 replicates of jackknife resampling, with tree search parameters set to 100 replicates, employing TBR branch swapping and holding up to 10 optimal trees. For BI, runs for the molecular data were performed under independent GTR+Γ+I models after partitioning by gene and codon position. Morphological data were run under the Mk+Γ model, with correction for ascertainment bias. MrBayes 3.2.2 [172], running 4 chains of Metropolis-coupled Markov-chain Monte Carlo for either 50 million generations (morphological and molecular datasets) or for 300 million generations (combined dataset), storing every 10,000^th^ generation and discarding the initial 25% of samples as burn-in. In every case, four independent runs were performed and the posterior samples were combined after confirming stationarity and convergence by examining traces and posterior distributions of parameters using Tracer v. 1.6 [173], as well as treespace exploration with R package *rwty* [174]. Gene trees were inferred under ML in PAUP* 4.0 [175] using the optimal model of evolution for each according to the Bayesian Information Criterion, as determined by that program. Concatenated molecular and morphological topologies were compared using SPR distances (i.e., the complement of the number of subtree-pruning and re-grafting moves required to convert one topology into the other divided by the maximum number of moves possible given the number of taxa), as implemented in TNT v. 1.5

### Assessment of conflict between genes

Gene trees were imported into the R environment [176] where topological differences were measured as unweighted RF distances [177] using package *phytools* [178]. For a pair of topologies, RF distances represent the sum bipartitions present in each tree and absent from the other, divided by the total number of bipartitions in both. In order to account for the fact that different gene trees contained different terminals, distances were calculated only after pairs of trees were pruned to the set of shared tips. For reference, the Bayesian topologies for the morphological and concatenated molecular datasets were also included in the set of compared topologies. RF distances were then used to build a graphical representation of topological conflict [179] in the program TreeScaper [180]. Variability was condensed into two dimensions using singular value decomposition of the distance matrix, minimizing the curvilinear components analysis stress [181].

Although this method visually depicts the degree of conflict among members of a set of topologies, it does not reveal whether differences are concentrated in a particular region of the tree. For this purpose, gene trees were employed to build a supernetwork using SuperQ v. 1.1 [182]. This approach decomposes trees into bipartitions, and a supernetwork was built in which branch lengths were calculated as the frequency of bipartitions in the set of ML gene trees using SplitsTree [183].

The length of a MSA depends on both the number of sites sampled and the alignment procedure. The use of the length of the alignment as a proxy for the strength of sampling errors assumes that the effect of the latter is negligible, or at least constant across MSAs of different lengths. Although disentangling the contributions of these two factors is not straightforward, we checked the quality of the MSA using the programs trimAl v. 1.2 [99] (using the *strictplus* method), BMGE v. 1.12 [100], and Gblocks v. 0.91b [101] (for the last two, the proportion of gaps tolerated was increased to 0.5; for Gblocks the minimum number of sequences for both conserved and flank positions was set to half the number of sequences). If final length is in fact a product of alignment procedures, these programs should detect a higher proportion of poorly aligned positions as the length of the alignment increases. By using a variety of programs that differentially rely on the proportion of gaps, degree of conservation, residue similarity, and entropy-based metrics, we hoped to recover a true signal of poorly aligned positions.

To assess whether topological conflict across gene trees is the result of low levels of phylogenetic signal, alternative resolutions of the backbone were statistically compared using site likelihoods after performing a set of constrained tree searches in PAUP* (similar to the approach of Arcila et al. [95]). Specifically, all searches were performed after constraining monophyly of each of the main lizard clades, as well as Squamata as a whole. Under these conditions, there are 945 possible resolutions of the lizard backbone tree [184]. We simplified the problem by comparing, for each gene, only the trees with the highest likelihood out of: 1) 105 possible alternatives supporting the monophyly of Scleroglossa; 2) 45 supporting the monophyly of Toxicofera; and 3) 795 rejecting both. Site likelihoods for these three competing hypotheses were used to perform approximately unbiased (AU) tests [91] using CONSEL v. 0.1 [185]. The AU test employs multiscale bootstrap of site likelihoods to simultaneously compare multiple trees and assign *P*-values to them. All trees with *P* > 0.05 cannot be statistically rejected as the best explanation for the observed data, and are referred to as the confidence set of trees.

### Rates of evolution

In order to explore the informativeness of molecular data, we calculated the rate of evolution of individual characters using HyPhy [186]. Rates were calculated for both the protein-coding dataset of Reeder et al. [56] and the UCE dataset of Streicher and Wiens [62]. For the latter, calculations were automated using TAPIR [187], increasing to five the minimum number of taxa without gaps for a site to be considered informative. We employed the time-calibrated topology of Zheng and Wiens [32], pruned to the set of taxa represented in each dataset (for taxa identified only to genus, a random species was selected for each genus), and estimated the ML rate of evolution per site under the optimal model for each gene. PI profiles were then plotted using the R package *PhyInformR* [188]. This enabled us to assess the relative utility of a set of characters to resolve relationships at different timescales [138], based on a comparison between their estimated rates of evolution and a theoretical optimum rate that maximizes the probability of correct resolution at a specific point in time. Given a set of rates, the PI profile of a dataset is expected to increase as one moves further back in time, up until the point at which characters start to evolve too fast to allow accurate phylogenetic resolution. The PI profile then decays, evidencing the expected accumulation of noise in the data.

For the morphological data, rates of evolution were estimated using BayesTraits V2 [189] under the simplest possible model including one parameter describing transition rates between states (Mk1 model [166]). Polymorphic entries, accounting for 0.2% of the morphological dataset, were transformed to missing data before the analysis. A direct comparison of rates of evolution for morphological and molecular data estimated on the same phylogeny would be unfair, as this topology would be far from optimal for one dataset, resulting in overly high inferred rates of evolution. Therefore, rates for the morphological partition were estimated in a tree in which Iguania was placed as sister to all other lizards, but with the rest of the topology being identical to the BI molecular tree (S4 Fig). We caution that the rates inferred for the morphological dataset on this tree are likely to be overestimated, and accordingly its informativeness shallower than the one estimated on an optimal topology. Branch lengths for this tree were calculated using the molecular dataset in PAUP* under a GTR+Γ+I model, and the tree was transformed to ultrametric using penalized likelihood [190] in R package *ape* [191]. The optimum value of the rate-smoothing parameter was determined through twofold cross-validation.

PI profiles do not account for the probability that homoplastic site patterns result in misleading support for spurious resolutions, and is therefore expected to perform poorly when characters evolve at fast rates and internodes are short [102]. Therefore, we employed the signal and noise framework [103] to estimate the probabilities of correct, incorrect, and polytomous resolution of the deepest internodes in the lizard tree, according to each gene. This approach has been recently found to be an accurate predictor of a gene’s utility to resolve a given phylogenetic question [148]. Probabilities of resolution for each of the four branches connecting the major lizard clades across all genes were calculated using optimal models of evolution, as well as accounting for the lengths of all five branches in the phylogenetic quartet of interest [188, 192, 193].

These methods rely on the molecular tree being correct, and can give biased estimates of the rate of evolution if the tree is misspecified. Therefore, we further explored the impact of fast evolving characters on phylogenetic inference using the tree-independent methods TIGER [109] and OV [110]. The first of these is based on character congruence, given that slow-evolving characters are expected to partition terminals into subgroups that will show little disagreement with those generated by other sites, while the partitions defined by fast-evolving characters will likely be the result of noise and therefore show low repeatability and high disagreement [104, 194]. The second method is much simpler, and only relies on calculating the number of character-state matches relative to mismatches across terminals and independently for each site, with the expectation that fast-evolving characters will explore more of the state space and therefore have a reduced state-matching probability. We used the molecular alignment obtained after using trimAl, that eliminated 16.9% of the characters, all of which were either extremely noisy or had high proportions of gaps (S11 Fig). All characters were then ordered from slowest to fastest according to both methods, and a set of matrices were generated by successively eliminating the 500 fastest-evolving characters, therefore obviating the need to define a strict fast/slow cut-off. ML inference was performed on these matrices with RAxML v. 8.2.10 [195] through the CIPRES gateway [196], using a GTRCAT+I model. It should be noted that tree-independent methods have recently been shown to produce biased character exclusion [197, 198], and should therefore be interpreted in the context of the other results, as a complement to methods that are tree-aware and ML-based. However, we find that both methods, but especially OV, were accurate at identifying fast-evolving sites, selecting characters that were also among those with the highest rates of evolution as estimated with ML in the time-calibrated topology (S12 Fig).

### Systematic biases

Two possible systematic biases in the molecular matrix were explored, namely among-lineage rate variation and compositional heterogeneity. For the first of these, a tree was inferred from the complete molecular dataset using PhyloBayes MPI v. 1.7 [199] under the site-heterogenous CAT+GTR model [200], which better accommodates scenarios of rate variation across lineages. Two independent chains were run for 10,000 cycles, discarding the first 25% as burn-in, and combined after checking for convergence. We randomly subsampled 10% of the trees in the posterior distribution to calculate a root-to-tip distance for all terminals. Distances for terminals in each of the main lizard clades were averaged to obtain a mean root-to-tip distance per clade, thus obtaining an estimate of the relative amount of molecular evolution for each (similar to the relative rate test [201, 202]). Rates across clades were explored by plotting the interval containing 95% of calculated distances, and rate differences were considered significant if intervals did not overlap.

Compositional biases were assessed using BaCoCa v. 1.103 [203]. Nucleotide frequencies for all terminals were calculated and subjected to a Chi-square test of homogeneity, as well as used to explore different parameters such as GC content and skew values for nucleotide pairs [204], known to negatively affect phylogenetic inference. GC content describes the general nucleotidic composition of the double-stranded molecule of DNA, while skew values describe compositional differences between strands [204]. Significance of the GC content difference found between Iguania + Serpentes and the remaining lizards was tested using simulation-based phylogenetic ANOVA [205] in *phytools* [178], with 1,000 simulations.

## Supporting information

S1 Fig Optimal tree for the morphological dataset under maximum parsimony. Values along branches represent jackknife support.
(TIF)

S2 Fig Majority rule consensus tree of the Bayesian inference analysis for the morphological dataset. Values along branches represent posterior probabilities.
(TIF)

S3 Fig Optimal tree for the molecular dataset under maximum parsimony. Values along branches represent jackknife support.
(TIF)

S4 Fig Majority rule consensus tree of the Bayesian inference analysis for the molecular dataset. Values along branches represent posterior probabilities.
(TIF)

S5 Fig Optimal tree for the combined dataset under maximum parsimony. Values along branches represent jackknife support.
(TIF)

S6 Fig Majority rule consensus tree of the Bayesian inference analysis for the combined dataset. Values along branches represent posterior probabilities.
(TIF)

S7 Fig Gene tree topological incongruence as a function of node age. Gene support frequency (GSF) corresponds to the fraction of gene trees showing a node present in the concatenated, time-calibrated tree of Zheng & Wiens [32] out of the set of genes sampling all terminals in the corresponding clade. The red curve is a loess regression, and shows a strong decay in GSF for the oldest 7 nodes of the topology. These correspond to the four backbone nodes plus the nodes for Iguania, Scincoidea and Lacertoidea. The degree of conflict in the resolution of those 7 nodes is also clear in the supernetwork of Fig 2. White dots show regions of the tree resolved differently after the addition of morphological data, all of which are among the nodes with lowest GSF.
(TIF)

S8 Fig Negative relationship between gene length and alignment certainty. Three different methods were employed to eliminate poorly aligned positions (trimAl, BMGE and Gblocks). All three methods eliminated a proportion of sites per gene that significantly correlates with the length of the multiple-sequence alignment (MSA, top). The targeted positions are shown in the bottom: each column of the alignment is represented as a box, with yellow, orange and red colored boxes showing positions targeted by any one, two or three of the methods, respectively. Genes are also ordered according to length of the MSA, decreasing from top left to bottom right by column. Most genes with low alignment certainty cluster towards the left.
(TIF)

S9 Fig Phylogenetic informativeness profiles of individual genes. Profiles are arbitrarily subdivided into those peaking before (left, 61%) and after 150 Ma (right, 39%). Note however that the majority of the genes on the right still have informativeness peaks before the estimated time-frame in which the main lizard clades diverged. Only 3 profiles peak during or after the squamate radiation, of which only 1 supports a monophyletic Toxicofera.
(TIF)

S10 Fig Collapse of the backbone topology after eliminating a relatively small fraction of fast-evolving sites. Topologies correspond to the optimal trees found using RAxML after eliminating the fastest-evolving 2,500 sites according to OV (left) or 4,000 sites according to TIGER (right). In both cases part of the lizard backbone topology collapsed, leaving Anguimorpha, Iguania, Lacertoidea and Serpentes in an unresolved polytomy. Further matrix pruning led to the collapse of all backbone branches. No other type of topological change was found.
(TIF)

S11 Fig Properties of the positions eliminated by trimAl. Targeted characters were either highly noisy, as measured using Shannon entropy, or had very high proportion of gaps. Density of eliminated characters increases from blue to red. The elimination of these characters had no impact on topology or support values.
(TIF)

S12 Fig Accuracy of tree-independent methods (OV and TIGER) to estimate rate of evolution. All variable characters in the molecular dataset (after eliminating poorly-aligned positions with trimAl) were ordered according to increasing rates of evolution, as estimated using maximum likelihood in the time-calibrated topology. The frequency with which characters were selected in a sliding window of size 1000 by both OV (orange) and TIGER (green) was fitted using a kernel regression smoother. The first 2,500 and 4,000 characters selected by each of these methodologies (whose exclusion led to the collapse of parts of the backbone topology, see S10 Fig) are among the ones with the fastest ML rates of evolution. OV, although simpler than TIGER, seems to be more accurate, showing a steeper rise in frequency towards the fastest extreme. This difference in accuracy might be the reason why the 4,000 fastest characters according to TIGER had to be deleted in order to obtain the same result as with only the 2,500 fastest ones according to OV. Perfect identification of the fastest characters is shown in dashed lines. Ten replicates of random character selection are also shown, the expected value of which is simply the proportion of eliminated characters out of the total.
(TIF)

S1 Table Timescale of the squamate radiation, as estimated by multiple time-calibration studies. Total time is the time spanned between the age of crown Squamata and that of the most recent common ancestor of Anguimorpha with either Iguania or Serpentes, depending on the resolution of Toxicofera obtained by each study. Average internode is the total time divided by five, giving the average length of the internodes connecting major squamate clades. PL = Penalized likelihood; GEA = Gauthier et al. [29]; CON = Conrad [38]; MkA = asymmetric Mk model.
(DOCX)

S2 Table Number of genes trees (GS = gene support) showing the same resolution as found in the concatenated molecular and combined topologies, for nodes in which these two differ. Low values are a consequence of both A) missing data, and more frequently B) other topologies being also commonly recovered.
(DOCX)

S3 Table Probabilities of incorrect (QIHP), polytomous (QIPP) and correct (QIRP) resolution of the four internodes connecting the main lizard clades across all genes. QIPP are relatively low overall, a consequence of the high rates of molecular evolution. Across all four branches and 46 genes, QIHP values surpass QIRP, evidencing higher probabilities of incorrect resolutions. Clade names follow the terminology of Vidal & Hedges (2009).
(DOCX)

S1 File Morphological dataset. (NEX)

S2 File Molecular dataset. (NEX)

S3 File Morphological synapomorphies relevant to crown squamate backbone. (DOCX)

S4 File Maximum likelihood gene trees. (NEX)
