# Supplementary figures and images for: Noise and biases in genomic data may underlie radically different hypotheses for the position of Iguania within Squamata
Source: PLoS One. 2018 Aug 22;13(8):e0202729. doi: 10.1371/journal.pone.0202729 (PMC6105018; doi:10.1371/journal.pone.0202729)

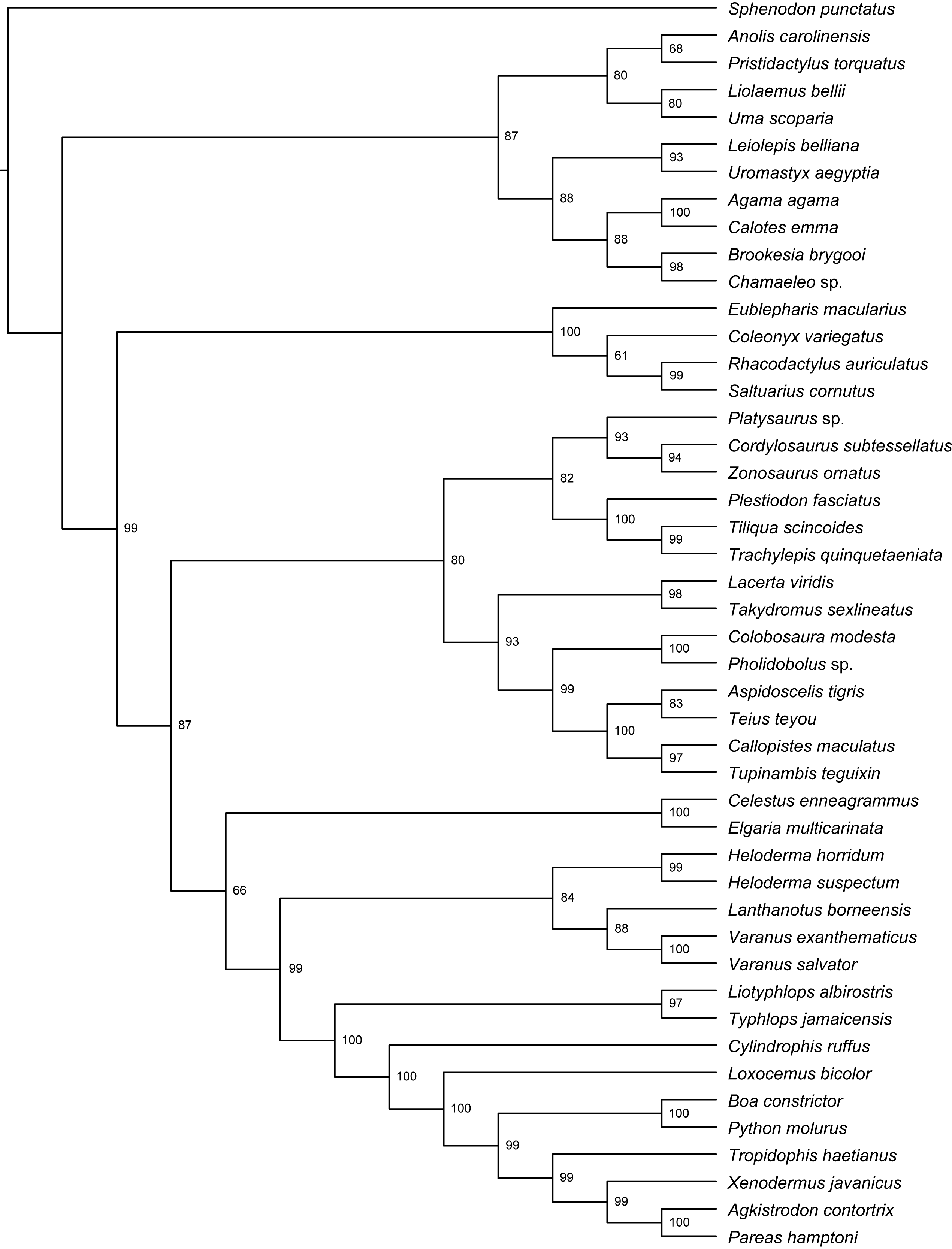

Supplement: S1 Fig — Values along branches represent jackknife support. (TIF) [file pone.0202729.s001.tif]

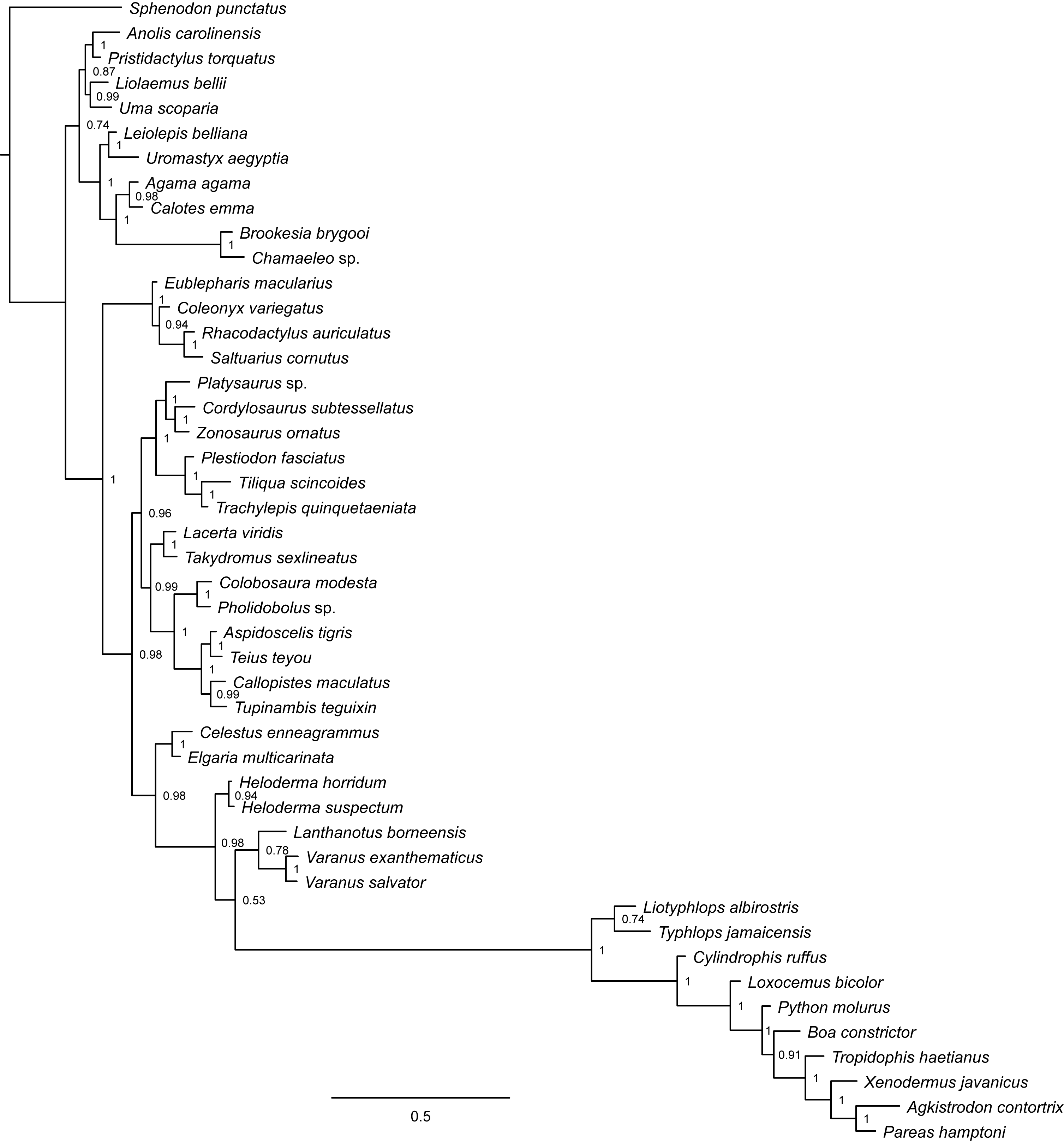

Supplement: S2 Fig — Values along branches represent posterior probabilities. (TIF) [file pone.0202729.s002.tif]

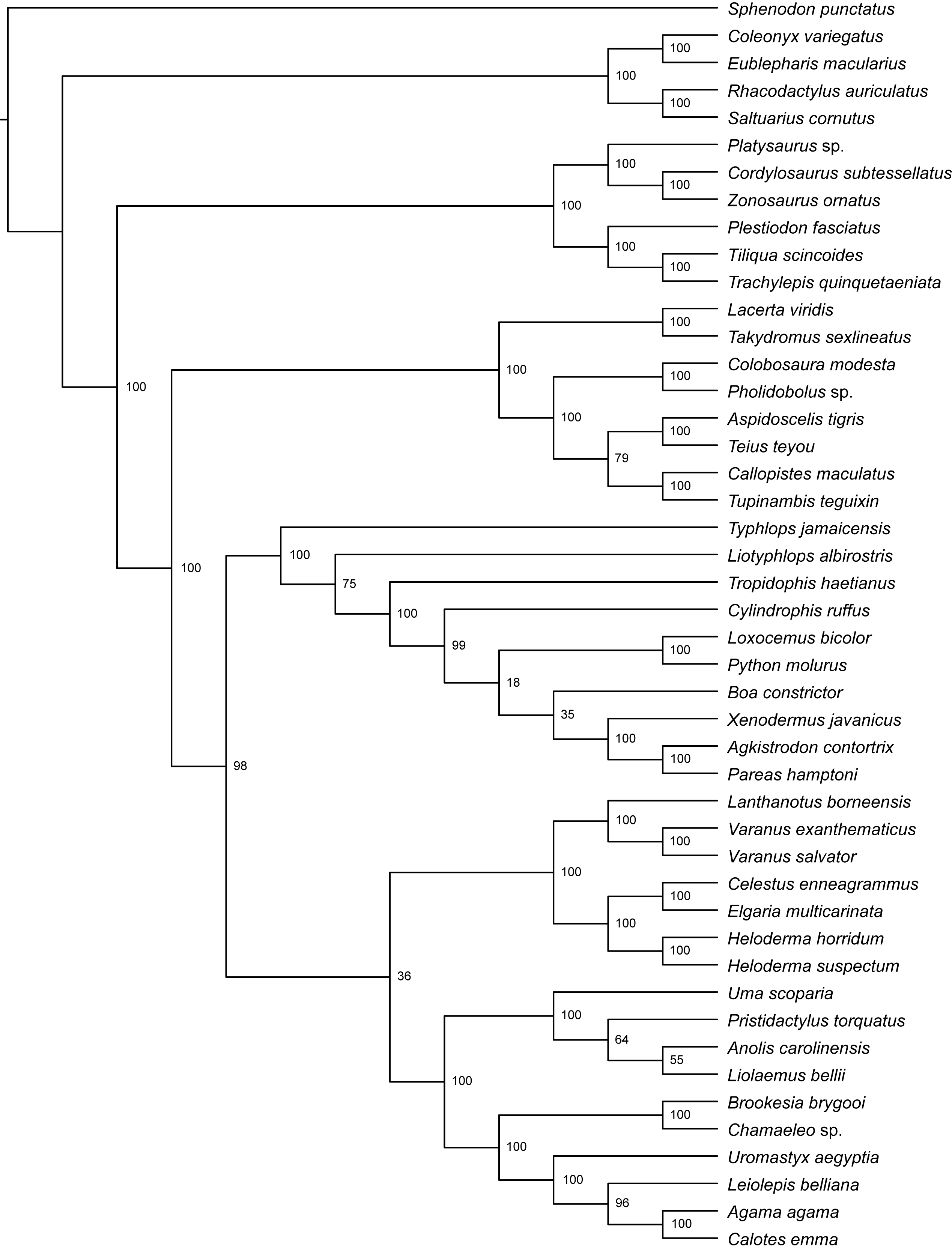

Supplement: S3 Fig — Values along branches represent jackknife support. (TIF) [file pone.0202729.s003.tif]

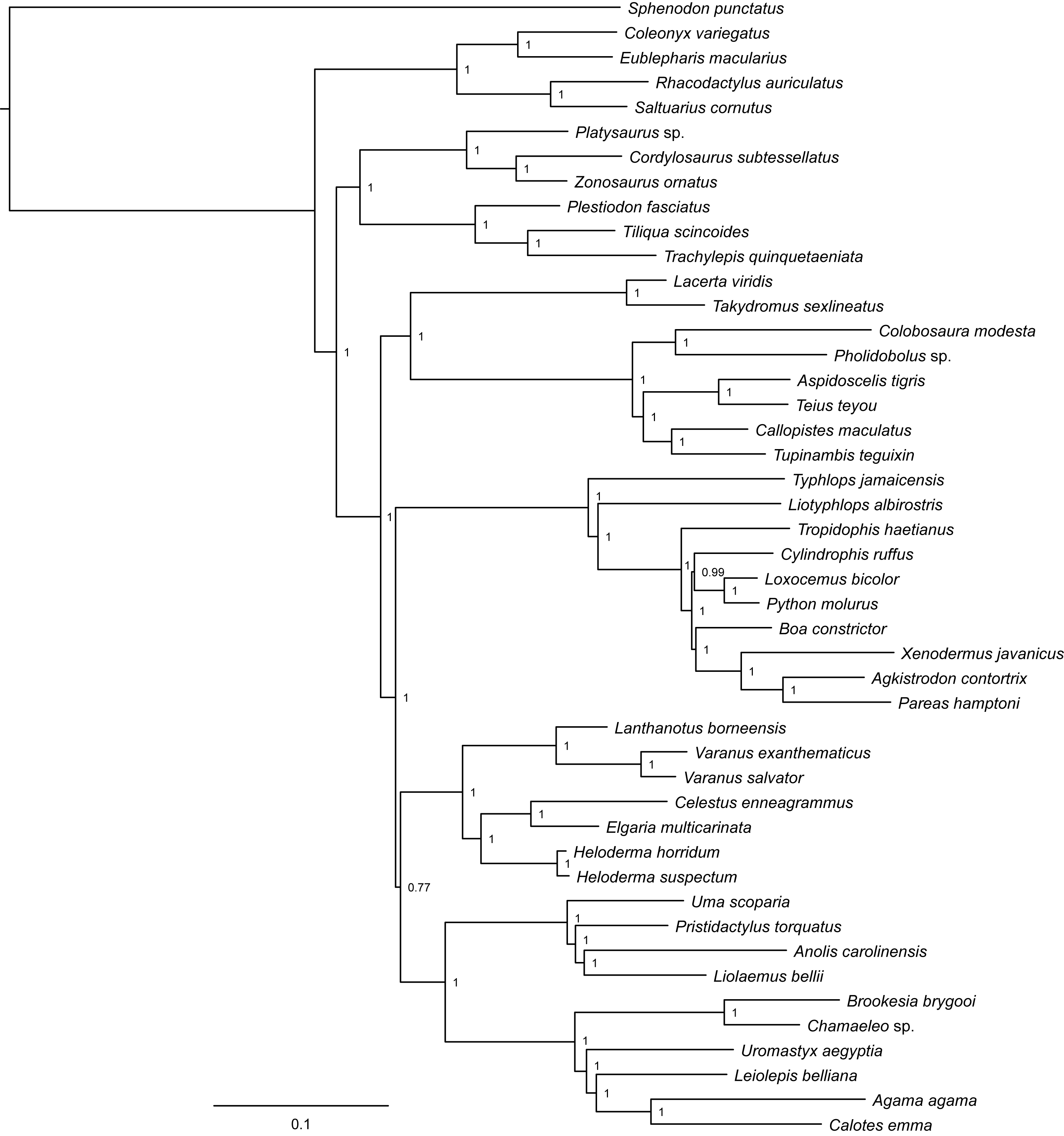

Supplement: S4 Fig — Values along branches represent posterior probabilities. (TIF) [file pone.0202729.s004.tif]

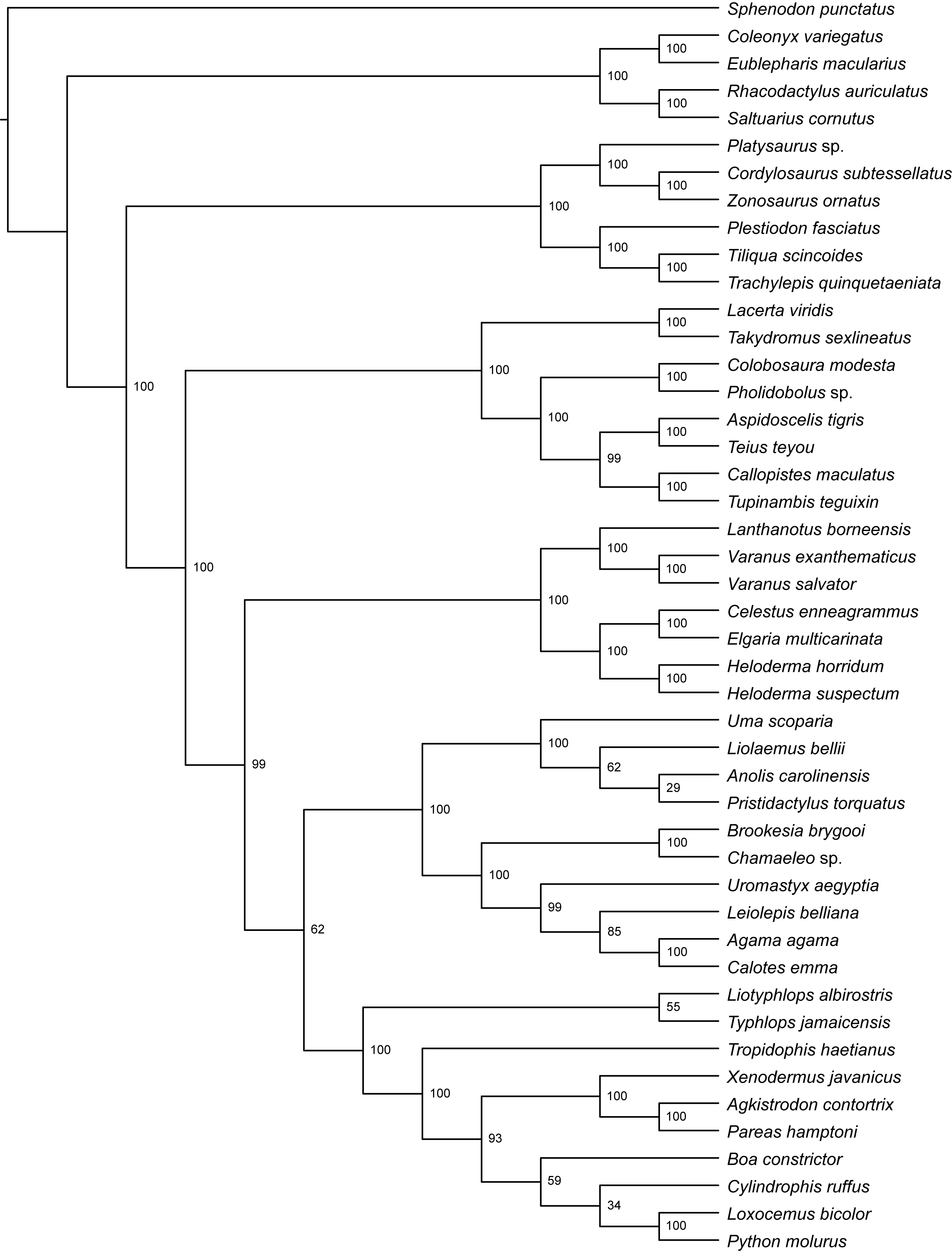

Supplement: S5 Fig — Values along branches represent jackknife support. (TIF) [file pone.0202729.s005.tif]

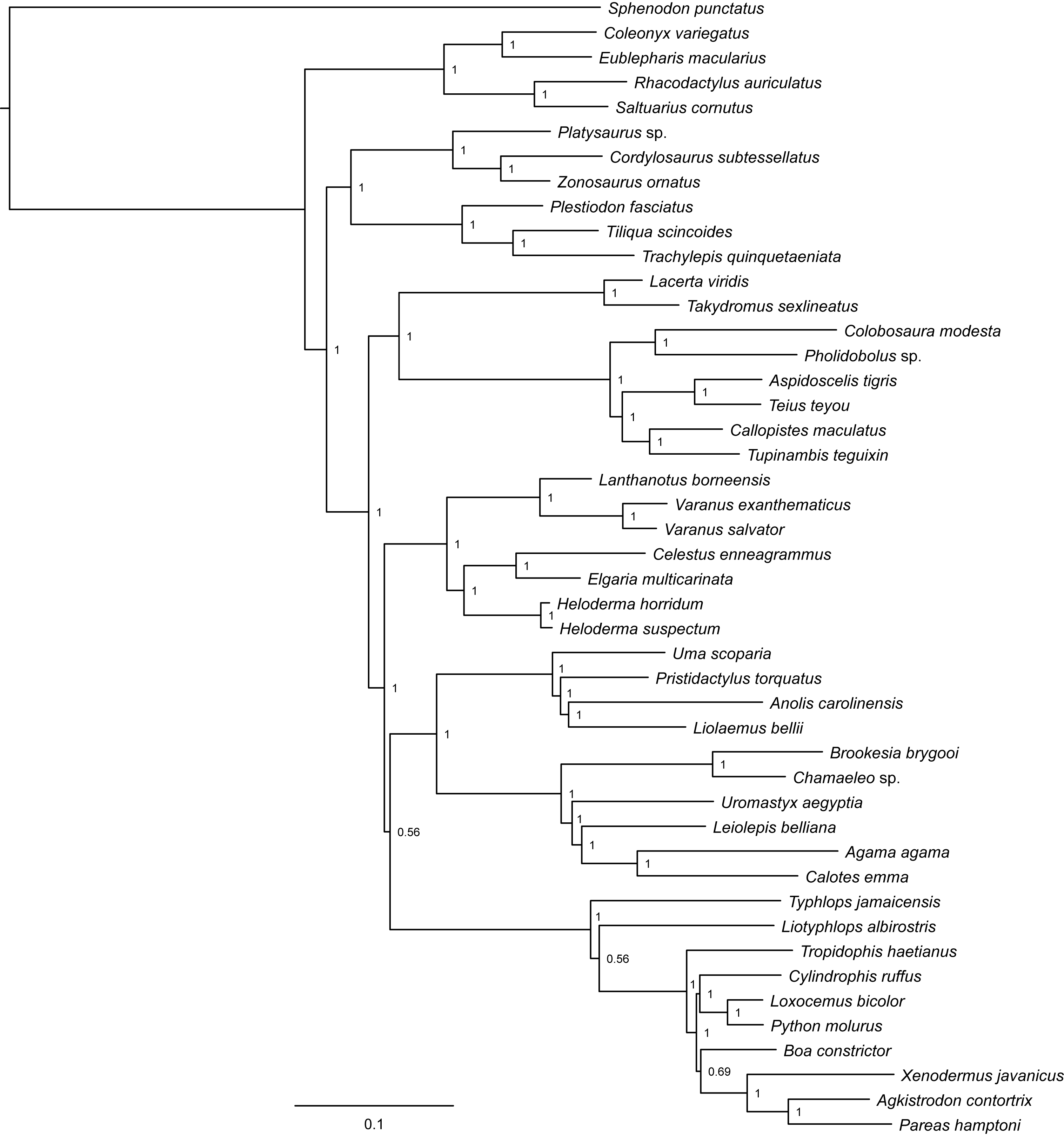

Supplement: S6 Fig — Values along branches represent posterior probabilities. (TIF) [file pone.0202729.s006.tif]

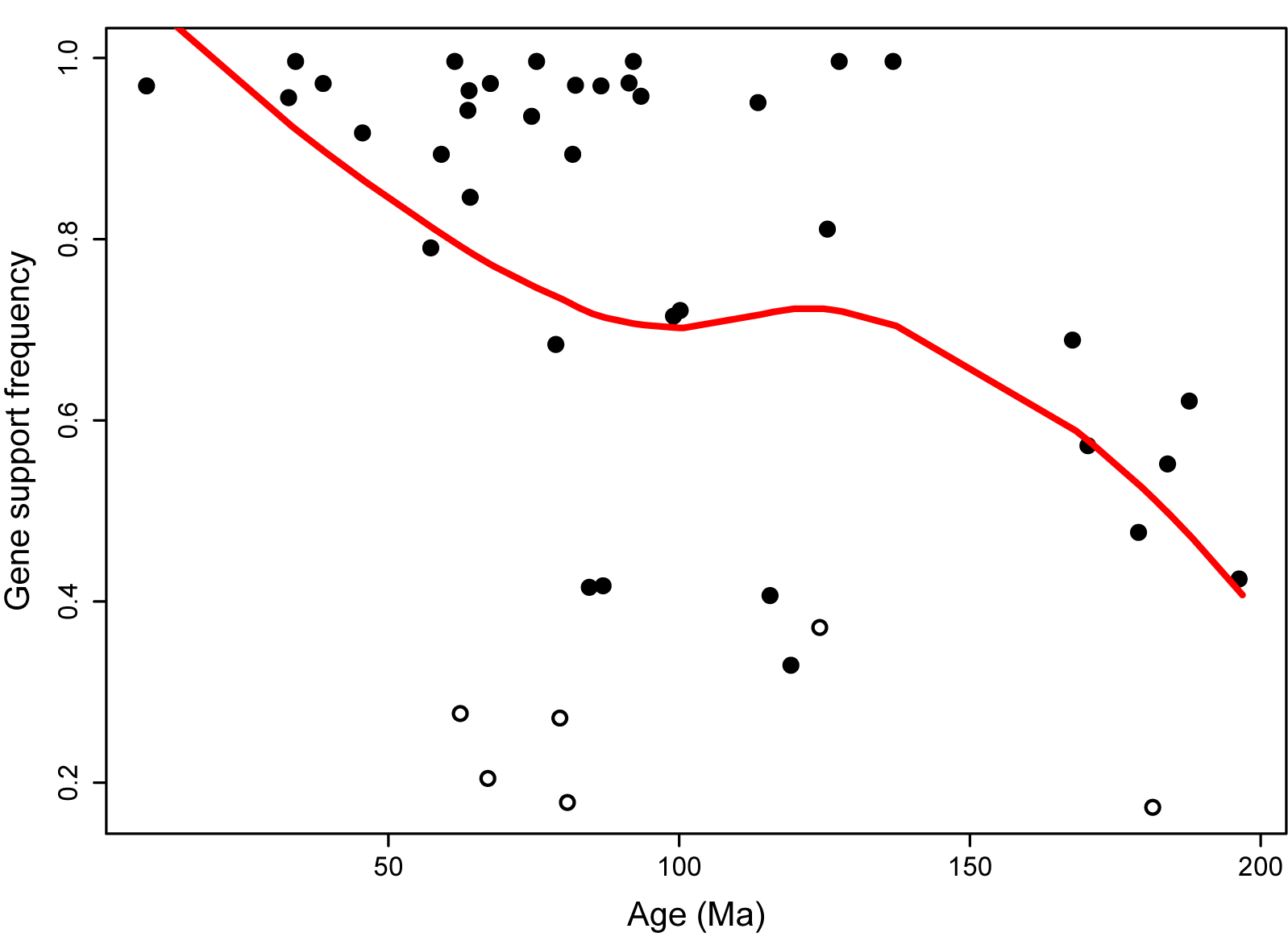

Supplement: S7 Fig — Gene support frequency (GSF) corresponds to the fraction of gene trees showing a node present in the concatenated, time-calibrated tree of Zheng & Wiens [32] out of the set of genes sampling all terminals in the corresponding clade. The red curve is a loess regression, and shows a strong decay in GSF for the oldest 7 nodes of the topology. These correspond to the four backbone nodes plus the nodes for Iguania, Scincoidea and Lacertoidea. The degree of conflict in the resolution of those 7 nodes is also clear in the supernetwork of Fig 2. White dots show regions of the tree resolved differently after the addition of morphological data, all of which are among the nodes with lowest GSF. (TIF) [file pone.0202729.s007.tif]

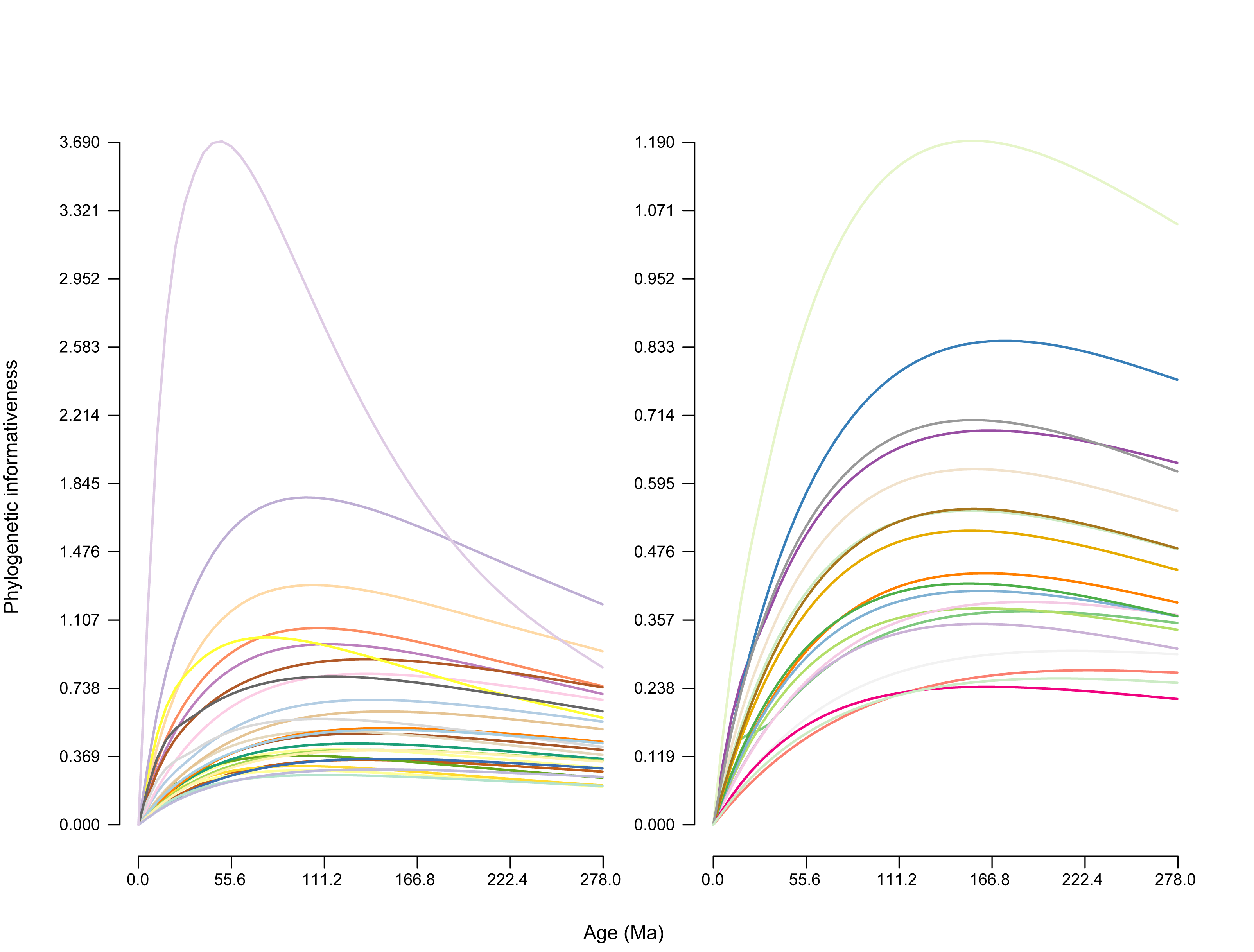

Supplement: S9 Fig — Profiles are arbitrarily subdivided into those peaking before (left, 61%) and after 150 Ma (right, 39%). Note however that the majority of the genes on the right still have informativeness peaks before the estimated time-frame in which the main lizard clades diverged. Only 3 profiles peak during or after the squamate radiation, of which only 1 supports a monophyletic Toxicofera. (TIF) [file pone.0202729.s009.tif]

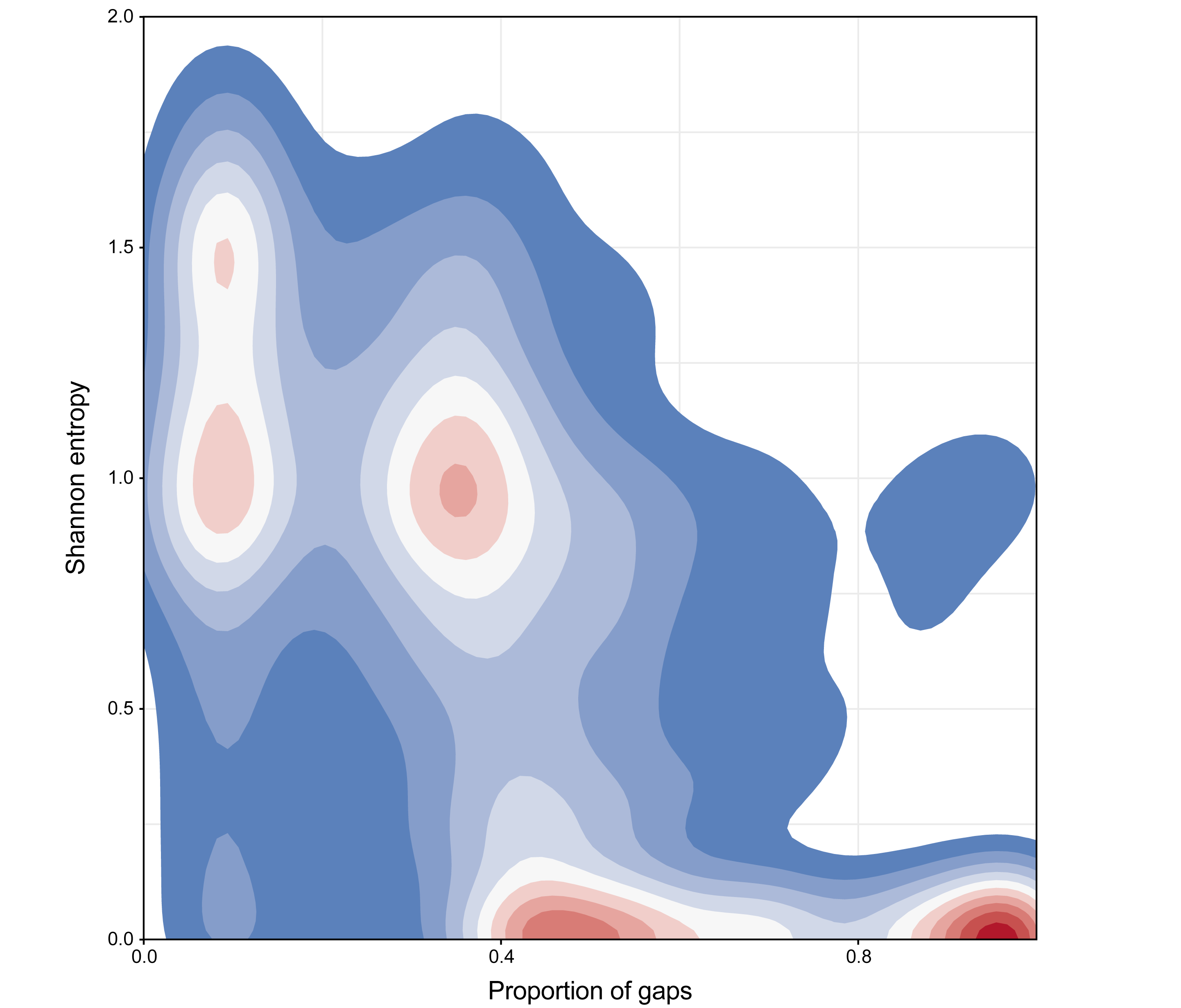

Supplement: S11 Fig — Targeted characters were either highly noisy, as measured using Shannon entropy, or had very high proportion of gaps. Density of eliminated characters increases from blue to red. The elimination of these characters had no impact on topology or support values. (TIF) [file pone.0202729.s011.tif]

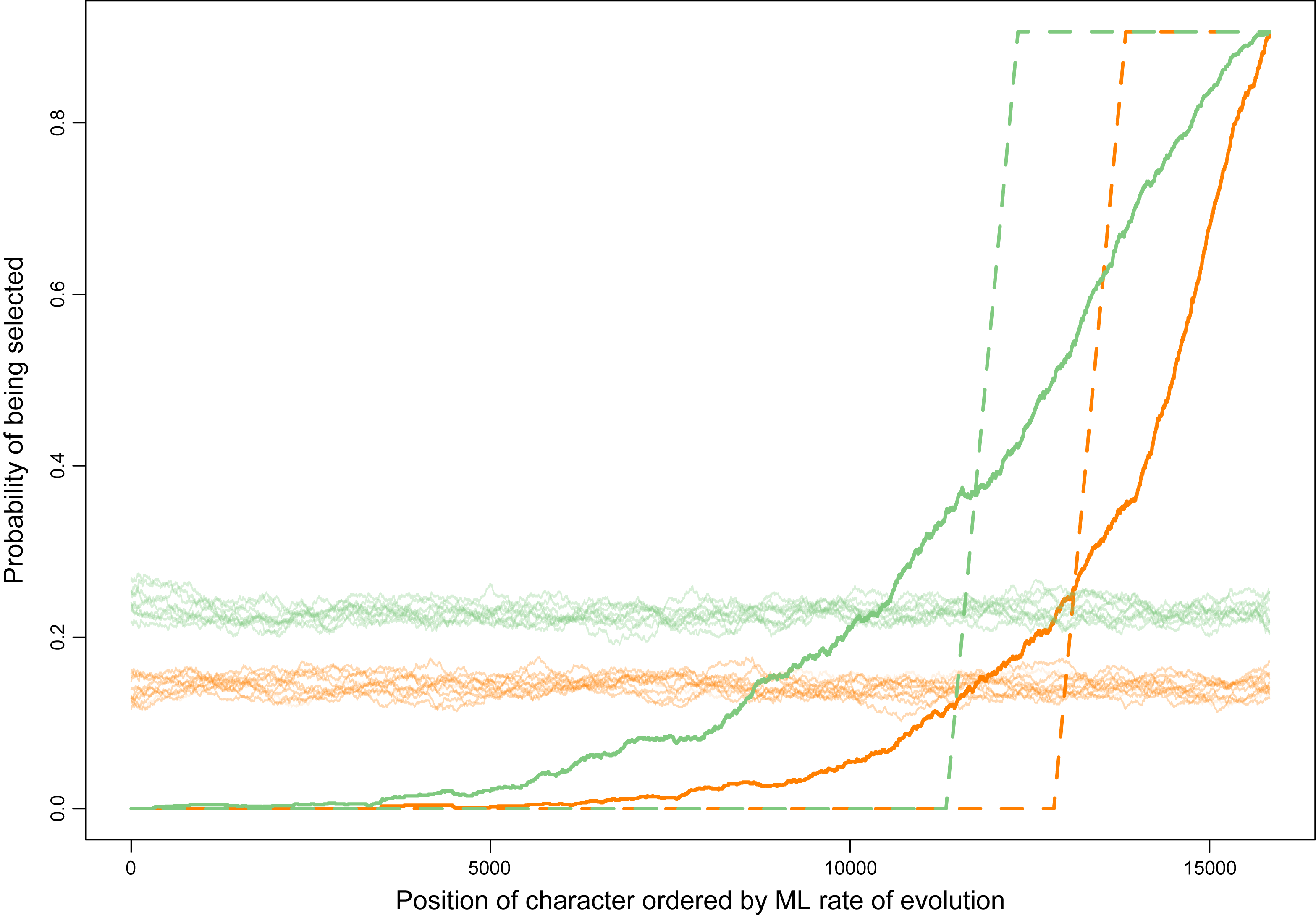

Supplement: S12 Fig — All variable characters in the molecular dataset (after eliminating poorly-aligned positions with trimAl) were ordered according to increasing rates of evolution, as estimated using maximum likelihood in the time-calibrated topology. The frequency with which characters were selected in a sliding window of size 1000 by both OV (orange) and TIGER (green) was fitted using a kernel regression smoother. The first 2,500 and 4,000 characters selected by each of these methodologies (whose exclusion led to the collapse of parts of the backbone topology, see S10 Fig) are among the ones with the fastest ML rates of evolution. OV, although simpler than TIGER, seems to be more accurate, showing a steeper rise in frequency towards the fastest extreme. This difference in accuracy might be the reason why the 4,000 fastest characters according to TIGER had to be deleted in order to obtain the same result as with only the 2,500 fastest ones according to OV. Perfect identification of the fastest characters is shown in dashed lines. Ten replicates of random character selection are also shown, the expected value of which is simply the proportion of eliminated characters out of the total. (TIF) [file pone.0202729.s012.tif]
