# Supplementary material for: Noise and biases in genomic data may underlie radically different hypotheses for the position of Iguania within Squamata
Source: PLoS One. 2018 Aug 22;13(8):e0202729. doi: 10.1371/journal.pone.0202729 (PMC6105018; doi:10.1371/journal.pone.0202729)
Supplement: S1 Table — Total time is the time spanned between the age of crown Squamata and that of the most recent common ancestor of Anguimorpha with either Iguania or Serpentes, depending on the resolution of Toxicofera obtained by each study. Average internode is the total time divided by five, giving the average length of the internodes connecting major squamate clades. PL = Penalized likelihood; GEA = Gauthier et al. [29]; CON = Conrad [38]; MkA = asymmetric Mk model. (DOCX) [file pone.0202729.s013.docx]

|  | Total time | Average internode length |
| --- | --- | --- |
| Mulcahy et al. (2012) PL | 24.1 | 4.8 |
| Mulcahy et al. (2012) BEAST | 43.5 | 8.7 |
| Jones et al. (2013) | 43 | 8.6 |
| Pyron & Burbrink (2014) | 28.1 | 5.6 |
| Zheng & Wiens (2016) | 23.1 | 4.6 |
| Pyron (2017) GEA combined MkA | 30.1 | 6 |
| Pyron (2017) CON combined | 29.7 | 5.9 |
