# Supplementary material for: Noise and biases in genomic data may underlie radically different hypotheses for the position of Iguania within Squamata
Source: PLoS One. 2018 Aug 22;13(8):e0202729. doi: 10.1371/journal.pone.0202729 (PMC6105018; doi:10.1371/journal.pone.0202729)
Supplement: S2 Table — Low values are a consequence of both A) missing data, and more frequently B) other topologies being also commonly recovered. (DOCX) [file pone.0202729.s014.docx]

| **Clade present in molecular tree** | **GS** | **Clade present in combined tree** | **GS** |
| --- | --- | --- | --- |
| **Maximum parsimony** | | | |
| *Typhlops* (*Liotyphlops* (Alethinophidia)) | 12 | (*Typhlops* + *Liotyphlops*) (Alethinophidia) | 11 |
| *Boa* (*Caenophidia*) | 7 | *Boa* (*Cylindrophis*, *Python*, *Loxocemus*) | 5 |
| *Pristidactylus* (*Liolaemus*, *Anolis*) | 4 | *Liolaemus (Pristidactylus,* *Anolis*) | 1 |
| **Maximum parsimony and Bayesian inference** | | | |
| Anguimorpha + Iguania | 7 | Serpentes + Iguania | 7 |
