# Supplementary material for: Noise and biases in genomic data may underlie radically different hypotheses for the position of Iguania within Squamata
Source: PLoS One. 2018 Aug 22;13(8):e0202729. doi: 10.1371/journal.pone.0202729 (PMC6105018; doi:10.1371/journal.pone.0202729)
Supplement: S3 Table — QIPP are relatively low overall, a consequence of the high rates of molecular evolution. Across all four branches and 46 genes, QIHP values surpass QIRP, evidencing higher probabilities of incorrect resolutions. Clade names follow the terminology of Vidal & Hedges (2009). (DOCX) [file pone.0202729.s015.docx]

|  | **Unidentata** | | | **Episquamata** | | | **Toxicofera** | | | **Iguania + Anguimorpha** | | |
| --- | --- | --- | --- | --- | --- | --- | --- | --- | --- | --- | --- | --- |
|  | **QIHP** | **QIPP** | **QIRP** | **QIHP** | **QIPP** | **QIRP** | **QIHP** | **QIPP** | **QIRP** | **QIHP** | **QIPP** | **QIRP** |
| **ADNP** | 0.519 | 0.123 | 0.358 | 0.436 | 0.134 | 0.431 | 0.523 | 0.136 | 0.341 | 0.543 | 0.138 | 0.319 |
| **AHR** | 0.545 | 0.106 | 0.350 | 0.478 | 0.115 | 0.408 | 0.549 | 0.115 | 0.336 | 0.566 | 0.116 | 0.318 |
| **CAND1** | 0.535 | 0.126 | 0.339 | 0.467 | 0.139 | 0.393 | 0.536 | 0.141 | 0.323 | 0.552 | 0.142 | 0.306 |
| **DLL1** | 0.553 | 0.112 | 0.335 | 0.499 | 0.121 | 0.380 | 0.557 | 0.121 | 0.323 | 0.571 | 0.121 | 0.308 |
| **ECEL** | 0.548 | 0.113 | 0.339 | 0.489 | 0.122 | 0.389 | 0.552 | 0.122 | 0.326 | 0.567 | 0.122 | 0.310 |
| **ENC1** | 0.548 | 0.101 | 0.351 | 0.482 | 0.110 | 0.408 | 0.552 | 0.110 | 0.337 | 0.569 | 0.111 | 0.320 |
| **GHSR** | 0.554 | 0.129 | 0.317 | 0.509 | 0.138 | 0.353 | 0.556 | 0.137 | 0.306 | 0.567 | 0.138 | 0.294 |
| **GPR37** | 0.561 | 0.113 | 0.326 | 0.515 | 0.121 | 0.364 | 0.564 | 0.120 | 0.316 | 0.576 | 0.121 | 0.303 |
| **INHIBA** | 0.552 | 0.096 | 0.352 | 0.488 | 0.104 | 0.408 | 0.557 | 0.104 | 0.339 | 0.573 | 0.104 | 0.322 |
| **LRRN1** | 0.551 | 0.110 | 0.338 | 0.493 | 0.120 | 0.387 | 0.554 | 0.120 | 0.326 | 0.569 | 0.121 | 0.310 |
| **LZTSS1** | 0.551 | 0.102 | 0.347 | 0.489 | 0.110 | 0.401 | 0.556 | 0.109 | 0.335 | 0.572 | 0.110 | 0.318 |
| **MLL3** | 0.544 | 0.072 | 0.385 | 0.462 | 0.076 | 0.462 | 0.553 | 0.076 | 0.370 | 0.575 | 0.077 | 0.348 |
| **NGFB** | 0.530 | 0.113 | 0.357 | 0.452 | 0.123 | 0.425 | 0.534 | 0.125 | 0.341 | 0.553 | 0.126 | 0.320 |
| **PRLR** | 0.549 | 0.075 | 0.375 | 0.473 | 0.081 | 0.446 | 0.557 | 0.081 | 0.362 | 0.577 | 0.081 | 0.342 |
| **PTGER4** | 0.551 | 0.132 | 0.317 | 0.503 | 0.142 | 0.356 | 0.553 | 0.141 | 0.306 | 0.564 | 0.142 | 0.294 |
| **PTPN** | 0.521 | 0.101 | 0.378 | 0.428 | 0.109 | 0.464 | 0.526 | 0.111 | 0.362 | 0.550 | 0.112 | 0.338 |
| **SINCAIP** | 0.544 | 0.116 | 0.340 | 0.482 | 0.126 | 0.392 | 0.547 | 0.126 | 0.326 | 0.563 | 0.127 | 0.310 |
| **NTF-3** | 0.542 | 0.108 | 0.350 | 0.473 | 0.118 | 0.409 | 0.546 | 0.118 | 0.336 | 0.563 | 0.119 | 0.318 |
| **SLC30A1** | 0.530 | 0.122 | 0.348 | 0.454 | 0.133 | 0.413 | 0.532 | 0.134 | 0.334 | 0.550 | 0.136 | 0.314 |
| **FSHR** | 0.549 | 0.101 | 0.350 | 0.486 | 0.109 | 0.406 | 0.555 | 0.108 | 0.337 | 0.571 | 0.109 | 0.320 |
| **ZEB2** | 0.532 | 0.116 | 0.352 | 0.458 | 0.127 | 0.415 | 0.536 | 0.128 | 0.336 | 0.554 | 0.130 | 0.316 |
| **MKL1** | 0.538 | 0.075 | 0.387 | 0.452 | 0.080 | 0.468 | 0.547 | 0.081 | 0.372 | 0.570 | 0.081 | 0.348 |
| **TRAF6** | 0.542 | 0.098 | 0.361 | 0.468 | 0.106 | 0.426 | 0.547 | 0.107 | 0.346 | 0.566 | 0.107 | 0.327 |
| **PNN** | 0.524 | 0.081 | 0.395 | 0.429 | 0.086 | 0.485 | 0.535 | 0.088 | 0.378 | 0.560 | 0.088 | 0.352 |
| **R35** | 0.539 | 0.088 | 0.373 | 0.460 | 0.095 | 0.446 | 0.546 | 0.095 | 0.358 | 0.567 | 0.096 | 0.337 |
| **SLC8A1** | 0.550 | 0.091 | 0.358 | 0.483 | 0.099 | 0.418 | 0.556 | 0.099 | 0.345 | 0.573 | 0.100 | 0.327 |
| **SLC8A3** | 0.547 | 0.087 | 0.366 | 0.475 | 0.095 | 0.430 | 0.554 | 0.095 | 0.351 | 0.572 | 0.096 | 0.332 |
| **VCPIP1** | 0.548 | 0.105 | 0.347 | 0.485 | 0.114 | 0.401 | 0.552 | 0.114 | 0.333 | 0.568 | 0.115 | 0.317 |
| **CARD4** | 0.537 | 0.077 | 0.386 | 0.451 | 0.083 | 0.467 | 0.546 | 0.083 | 0.371 | 0.569 | 0.084 | 0.347 |
| **MSH6** | 0.545 | 0.088 | 0.367 | 0.471 | 0.095 | 0.434 | 0.552 | 0.095 | 0.353 | 0.571 | 0.096 | 0.333 |
| **HLCS** | 0.543 | 0.088 | 0.369 | 0.467 | 0.095 | 0.438 | 0.550 | 0.096 | 0.355 | 0.570 | 0.096 | 0.334 |
| **GALR1** | 0.541 | 0.141 | 0.318 | 0.487 | 0.152 | 0.361 | 0.542 | 0.152 | 0.306 | 0.555 | 0.154 | 0.292 |
| **CXCR4** | 0.548 | 0.097 | 0.355 | 0.481 | 0.104 | 0.414 | 0.554 | 0.104 | 0.342 | 0.571 | 0.105 | 0.324 |
| **CILP** | 0.534 | 0.063 | 0.403 | 0.441 | 0.067 | 0.492 | 0.546 | 0.068 | 0.386 | 0.572 | 0.068 | 0.360 |
| **AKAP9** | 0.526 | 0.069 | 0.405 | 0.426 | 0.074 | 0.500 | 0.537 | 0.075 | 0.387 | 0.564 | 0.076 | 0.360 |
| **BACH1** | 0.519 | 0.064 | 0.417 | 0.411 | 0.068 | 0.521 | 0.531 | 0.070 | 0.399 | 0.561 | 0.071 | 0.369 |
| **BDNF** | 0.536 | 0.137 | 0.327 | 0.475 | 0.149 | 0.376 | 0.537 | 0.150 | 0.313 | 0.551 | 0.152 | 0.297 |
| **BHLHB2** | 0.542 | 0.097 | 0.361 | 0.470 | 0.105 | 0.425 | 0.548 | 0.106 | 0.346 | 0.567 | 0.106 | 0.327 |
| **BMP2** | 0.547 | 0.106 | 0.347 | 0.483 | 0.115 | 0.402 | 0.552 | 0.115 | 0.333 | 0.568 | 0.116 | 0.317 |
| **ZFP36L1** | 0.545 | 0.122 | 0.333 | 0.486 | 0.133 | 0.381 | 0.548 | 0.133 | 0.320 | 0.562 | 0.134 | 0.304 |
| **UBN1** | 0.559 | 0.075 | 0.366 | 0.494 | 0.080 | 0.426 | 0.567 | 0.079 | 0.354 | 0.585 | 0.079 | 0.336 |
| **RAG1** | 0.539 | 0.082 | 0.379 | 0.456 | 0.089 | 0.455 | 0.547 | 0.090 | 0.364 | 0.568 | 0.090 | 0.342 |
| **NKTR** | 0.536 | 0.056 | 0.407 | 0.442 | 0.059 | 0.499 | 0.549 | 0.060 | 0.391 | 0.575 | 0.060 | 0.365 |
| **FSTL5** | 0.537 | 0.125 | 0.338 | 0.472 | 0.138 | 0.391 | 0.539 | 0.138 | 0.322 | 0.555 | 0.140 | 0.305 |
| **cMos** | 0.550 | 0.078 | 0.372 | 0.477 | 0.084 | 0.440 | 0.558 | 0.084 | 0.359 | 0.577 | 0.084 | 0.339 |
| **ND2** | 0.573 | 0.051 | 0.376 | 0.512 | 0.053 | 0.434 | 0.583 | 0.052 | 0.365 | 0.600 | 0.052 | 0.348 |
