# Supplementary material for: Noise and biases in genomic data may underlie radically different hypotheses for the position of Iguania within Squamata
Source: PLoS One. 2018 Aug 22;13(8):e0202729. doi: 10.1371/journal.pone.0202729 (PMC6105018; doi:10.1371/journal.pone.0202729)
Supplement: S3 File — (DOCX) [file pone.0202729.s018.docx]

**Morphological synapomorphies relevant to backbone diversification of**

**crown Squamata**

A total of 64 unambiguous synapomorphies support monophyly of Scleroglossa (26), Autarchoglossa (18), and Anguimorpha (including Serpentes) (20) to the exclusion of Iguania. They are listed below, with a two-letter abbreviation corresponding to the anatomical system they are assigned to, and with references to published images of qualitative character states. A key to the abbreviations and some descriptive statistics indicating the range of systems surveyed, including their relative contributions to these hypotheses, are provided below.

In the context of our more comprehensive analysis - to be published in full elsewhere - a few of these putative synapomorphies may be sampling artifacts (e.g., Scleroglossa synapomorphy 16) and intermediate stem fossils will reduce the number of synapomorphies accumulating at crown nodes (e.g., many of the putative scleroglossan synapomorphies are present in stem scleroglossans such as extinct Polyglyphanodontia). A reference to published figures showing character states is included when available.

**Scleroglossa**:

| 1) | DC* | Frontal subolfactory process > 41% of depth from skull roof to palatine |
| --- | --- | --- |
| 2) | MU | Temporal muscles originate ventrally on parietal table and supratemporal process (Gauthier et al., 2012: fig. 90) |
| 3) | DC | Maxilla facial process length/maxilla length > 37% |
| 4) | DC | Prefrontal orbitonasal margin slopes ventromedially (Gauthier et al., 2012: fig. 128) |
| 5) | DC | Squamosal temporal process base lies against parietal (Gauthier et al., 2012: fig. 1162) |
| 6) | DC | Squamosal ascending process absent (Gauthier et al., 2012: fig. 165) |
| 7) | DC | Squamosal distinct transverse facet for quadrate head absent (new character) |
| 8) | DC | Quadrate slopes anteroventrally (> 121°) |
| 9) | DC* | Septomaxilla medial flange present (Gauthier et al., 2012: fig. 205) |
| 10) | NV* | Nervus ethmoidalis medialis enclosed in septomaxilla anteriorly (Gauthier et al., 2012: fig. 208) |
| 11) | DC | Palatine choanal fossa well-developed, extending about half way back on palatine (Gauthier et al., 2012: fig. 250) |
| 12) | DC | Ectopterygoid obtuse angle between palatal and maxillary rami (Gauthier et al., 2012: fig. 272) |
| 13) | DC | Ectopterygoid posterior process reduced to no more than small lateral knob (Gauthier et al., 2012: fig. 283) |
| 14) | DC | Ectopterygoid dorsal process reduced/absent (Gauthier et al., 2012: fig. 285) |
| 15) | CC | Prootic alar process present (Gauthier et al., 2012: fig. 305) |
| 16) | DC | Dentary coronoid process extends no further posteriorly than to apex of coronoid bone (Gauthier et al., 2012: fig. 364) |
| 17) | DE | Median premaxillary tooth present (Gauthier et al., 2012: fig. 413) |
| 18) | DE | Premaxillary tooth count > 6 |
| 19) | HY | Basihyal extends ventral to level of braincase when mouth is closed (Gauthier et al., 2012: fig. 439) |
| 20) | VC | Presacral vertebral count > 25 |
| 21) | LG | Ilium sacral rib attachments entirely posterior to level of acetabulum (new character) |
| 22) | LG | Tibia with notched distal epiphysis attached to complementary ridge on astragalus (Gauthier et al., 2012: fig. 555) |
| 23) | TO* | Foretongue chemosensory portion covered with flattened papillae (Gauthier et al., 2012: fig. 600) |
| 24) | BE* | Lingual prey-prehension absent |
| 25) | SC | Rostral scale contacts nasal scale |
| 26) | SC | Nasal scale contacts supralabial scales |

**Autarchoglossa:**

| 1) | DC | Nasal supranarial process reduced/absent (Gauthier et al., 2012: fig. 22) |
| --- | --- | --- |
| 2) | DC* | Frontal subolfactory process > 57% of depth from skull roof to palatine |
| 3) | LD | Maxilla with naso-lacrimal fossa internally (Gauthier et al., 2012: fig. 117) |
| 4) | DC | Jugal extends ventral to lacrimal bone (Gauthier et al., 2012: fig. 145) |
| 5) | DC* | Septomaxilla contacts dorsal surface of palatal shelf of maxilla (Gauthier et al., 2012: fig. 201) |
| 6) | NV* | Nervus ethmoidalis medialis enclosed in anterior half of septomaxilla (Gauthier et al., 2012: fig. 208) |
| 7) | DC* | Vomer encapsulates vomeronasal organ posteriorly and medially (Gauthier et al., 2012: fig. 217) |
| 8) | NV* | Vomeronasal nerve lies in canal on vomer posterodorsally (Gauthier et al., 2012: fig. 220) |
| 9) | NV | Infraorbital canal enclosed entirely in palatine bone (Gauthier et al., 2012: fig. 245) |
| 10) | DC | Ectopterygoid with slot-like maxilla articulation (Gauthier et al., 2012: fig. 275) |
| 11) | NV | Posterior auditory foramen enclosed entirely in prootic (Gauthier et al., 2012: fig. 315) |
| 12) | DC | Splenial extends anteriorly to about one-half length of dentary tooth row (Gauthier et al., 2012: fig. 375) |
| 13) | DE | Dentary teeth directed dorsolaterally (new character) |
| 14) | VC | Caudal autotomic septum within caudal rib (Gauthier et al., 2012: fig. 470) |
| 15) | LG | Clavicle strongly angulated at mid-shaft away from scapulocoracoid (Gauthier et al., 2012: fig. 502) |
| 16) | MB | Dermal skull roof covered by rugose metaplastic bone (Gauthier et al., 2012: fig. 572) |
| 17) | MU | Rectus abdominis muscles insert on skin between transverse ventral scale rows (Gauthier et al., 2012: fig. 607) |
| 18) | SC | Supraocular scales contain single osteoderms (Gauthier et al., 2012: fig. 575) |

**Anguimorpha** (= Anguidae, Varanoidea, and Serpentes in this analysis):

| 1) | DC* | Frontal subolfactory process > 75% of depth between skull roof and palatine (Gauthier et al., 2012: fig. 39) |
| --- | --- | --- |
| 2) | LD | Lacrimal bone encloses most of lacrimal duct (Gauthier et al., 2012: fig. 138) |
| 3) | DC | Vomer with prominent ventral longitudinal ridge present, converging toward midline, and most developed posterior to level of vomeronasal nerve exit from Septomaxilla (Gauthier et al., 2012: fig. 222) |
| 4) | DC | Palatine vomerine process long, slender, finger-like tine clasped in groove on dorsal surface of vomer (Gauthier et al., 2012: fig. 234) |
| 5) | NV | Vidian canal caudal aperture near sphenoid-prootic suture (Gauthier et al., 2012: fig. 337) |
| 6) | MB | Apophysial ossification caps basal tuber (Gauthier et al., 2012: fig. 340) |
| 7) | DC | Dentary posterior end on lateral face of mandible below or anterior to level of coronoid apex (Gauthier et al., 2012: fig. 369) |
| 8) | DC | Dentary Meckel’s canal opens ventrally cranial to anterior inferior alveolar foramen (Gauthier et al., 2012: fig. 371) |
| 9) | NV | Posterior mylohyoid foramen set anterior to level of coronoid apex (Gauthier et al., 2012: fig. 385) |
| 10) | DC | Retroarticular process rotates about long axis so that medial margin is lower than the lateral margin (new character) |
| 11) | DE | Replacement teeth arise posterolingually (Gauthier et al., 2012: fig. 428) |
| 12) | HY | Second ceratobranchial in hyoid apparatus absent (Gauthier et al., 2012: fig. 446) |
| 13) | VC | Vertebral column lengthens to > 27 presacrals |
| 14) | VC | Zygosphene-zygantrum intervertebral joint absent (Gauthier et al., 2012: fig. 468) (strongly developed in snakes) |
| 15) | VC | Caudal haemal arch (intercentrum) sutured to pedicles on posterior margin of preceding centrum (Gauthier et al., 2012: fig. 475) |
| 16) | MB | Osteoderms in gular scales (Gauthier et al., 2012: fig. 578) (absent in snakes) |
| 17) | MB | Osteoderms in dorsal scales (Gauthier et al., 2012: fig. 579) (absent in snakes) |
| 18) | TO* | Foretongue retracts into hind tongue (Gauthier et al., 2012: fig. 589) |
| 19) | TO* | Hind tongue papilla sharply pointed (Gauthier et al., 2012: fig. 603) |
| 20) | SC | Frontoparietal scales reduced, contact on midline absent, separated by contact between frontal and interparietal scales |

**Key to anatomical systems (and behavior) and their relative contributions to phenomic dataset**

|  | **Number of synapomorphies** | **Percentage of total** |
| --- | --- | --- |
| (DC) Dermocranium | 26 | 41 |
| (CC) Chondrocranium | 1 | 2 |
| (NV) Neurovascular system | 7 | 11 |
| (LD) Lacrimal duct | 2 | 3 |
| (DE) Dentition | 3 | 5 |
| (HY) Hyoid apparatus | 2 | 3 |
| (VC) Vertebral column | 5 | 8 |
| (LG) Limbs & girdles | 3 | 5 |
| (MB) Metaplastic bone | 4 | 6 |
| (MU) Musculature | 2 | 3 |
| (SC) Scutellation | 5 | 8 |
| (TO) Tongue | 3 | 5 |
| (BE) Behavior | 1 | 2 |
| *Vomeronasal system | 13 | 20 |

**Skull** = 46 (72%) **Postcranium** = 17 (27%) **Behavioral** = 1 (2%)

**Distribution of backbone synapomorphies provided by the skull, subdivided by region**

|  | **Number of synapomorphies** | **Percentage of total** |
| --- | --- | --- |
| Preorbital | 7 | 18 |
| Orbital | 6 | 16 |
| Postorbital | 5 | 13 |
| Palate | 10 | 26 |
| Braincase | 4 | 11 |
| Mandible | 6 | 16 |

Morphological synapomorphies pertinent to phylogenetic relationships among backbone clades of crown Squamata derive from disparate functional, anatomical, and developmental systems. That being said, most of them come from the skull (72%), with an important contribution from the postcranium (27%). The complex dermocranium in particular has proven a rich source of data (41%). For the most part, backbone synapomorphies appear fairly evenly distributed across major skull regions. The tongue itself, often seen as the chief impediment to accepting the molecular topology, accounts for just 5% of the backbone synapomorphies marking the initial squamate radiation. But the tongue is just part of the sophisticated vomeronasal sensory system elaborating within Squamata — a system that accounts for 20% of all ‘backbone’ synapomorphies — but that remains in the unmodified ancestral condition in Iguania and in the squamate sister clade *Sphenodon* *punctatus*.

Gauthier, J.A., M. Kearney, J.A. Maisano, O. Rieppel, and A. Behlke. 2012. Assembling the squamate tree of life: perspectives from the phenotype and the fossil record. Bulletin of the Peabody Museum of Natural History 53(1) 3-308.
